# Gapless genome assembly of azalea and multi-omics investigation into divergence between two species with distinct flower color

**DOI:** 10.1093/hr/uhac241

**Published:** 2022-10-26

**Authors:** Shuai Nie, Shi-Wei Zhao, Tian-Le Shi, Wei Zhao, Ren-Gang Zhang, Xue-Chan Tian, Jing-Fang Guo, Xue-Mei Yan, Yu-Tao Bao, Zhi-Chao Li, Lei Kong, Hai-Yao Ma, Zhao-Yang Chen, Hui Liu, Yousry A El-Kassaby, Ilga Porth, Fu-Sheng Yang, Jian-Feng Mao

**Affiliations:** National Engineering Research Center of Tree Breeding and Ecological Restoration, Beijing Advanced Innovation Center for Tree Breeding by Molecular Design, Key Laboratory of Genetics and Breeding in Forest Trees and Ornamental Plants, Ministry of Education, College of Biological Sciences and Technology, Beijing Forestry University, Beijing 100083, China; National Engineering Research Center of Tree Breeding and Ecological Restoration, Beijing Advanced Innovation Center for Tree Breeding by Molecular Design, Key Laboratory of Genetics and Breeding in Forest Trees and Ornamental Plants, Ministry of Education, College of Biological Sciences and Technology, Beijing Forestry University, Beijing 100083, China; National Engineering Research Center of Tree Breeding and Ecological Restoration, Beijing Advanced Innovation Center for Tree Breeding by Molecular Design, Key Laboratory of Genetics and Breeding in Forest Trees and Ornamental Plants, Ministry of Education, College of Biological Sciences and Technology, Beijing Forestry University, Beijing 100083, China; Department of Ecology and Environmental Science, Umeå Plant Science Centre, Umeå University, SE-901 87 Umeå, Sweden; Department of Bioinformatics, Ori (Shandong) Gene Science and Technology Co., Ltd., Weifang 261322, China; National Engineering Research Center of Tree Breeding and Ecological Restoration, Beijing Advanced Innovation Center for Tree Breeding by Molecular Design, Key Laboratory of Genetics and Breeding in Forest Trees and Ornamental Plants, Ministry of Education, College of Biological Sciences and Technology, Beijing Forestry University, Beijing 100083, China; National Engineering Research Center of Tree Breeding and Ecological Restoration, Beijing Advanced Innovation Center for Tree Breeding by Molecular Design, Key Laboratory of Genetics and Breeding in Forest Trees and Ornamental Plants, Ministry of Education, College of Biological Sciences and Technology, Beijing Forestry University, Beijing 100083, China; National Engineering Research Center of Tree Breeding and Ecological Restoration, Beijing Advanced Innovation Center for Tree Breeding by Molecular Design, Key Laboratory of Genetics and Breeding in Forest Trees and Ornamental Plants, Ministry of Education, College of Biological Sciences and Technology, Beijing Forestry University, Beijing 100083, China; National Engineering Research Center of Tree Breeding and Ecological Restoration, Beijing Advanced Innovation Center for Tree Breeding by Molecular Design, Key Laboratory of Genetics and Breeding in Forest Trees and Ornamental Plants, Ministry of Education, College of Biological Sciences and Technology, Beijing Forestry University, Beijing 100083, China; National Engineering Research Center of Tree Breeding and Ecological Restoration, Beijing Advanced Innovation Center for Tree Breeding by Molecular Design, Key Laboratory of Genetics and Breeding in Forest Trees and Ornamental Plants, Ministry of Education, College of Biological Sciences and Technology, Beijing Forestry University, Beijing 100083, China; National Engineering Research Center of Tree Breeding and Ecological Restoration, Beijing Advanced Innovation Center for Tree Breeding by Molecular Design, Key Laboratory of Genetics and Breeding in Forest Trees and Ornamental Plants, Ministry of Education, College of Biological Sciences and Technology, Beijing Forestry University, Beijing 100083, China; National Engineering Research Center of Tree Breeding and Ecological Restoration, Beijing Advanced Innovation Center for Tree Breeding by Molecular Design, Key Laboratory of Genetics and Breeding in Forest Trees and Ornamental Plants, Ministry of Education, College of Biological Sciences and Technology, Beijing Forestry University, Beijing 100083, China; National Engineering Research Center of Tree Breeding and Ecological Restoration, Beijing Advanced Innovation Center for Tree Breeding by Molecular Design, Key Laboratory of Genetics and Breeding in Forest Trees and Ornamental Plants, Ministry of Education, College of Biological Sciences and Technology, Beijing Forestry University, Beijing 100083, China; National Engineering Research Center of Tree Breeding and Ecological Restoration, Beijing Advanced Innovation Center for Tree Breeding by Molecular Design, Key Laboratory of Genetics and Breeding in Forest Trees and Ornamental Plants, Ministry of Education, College of Biological Sciences and Technology, Beijing Forestry University, Beijing 100083, China; Department of Forest and Conservation Sciences, Faculty of Forestry, University of British Columbia, Vancouver, BC, V6T 1Z4, Canada; Départment des Sciences du Bois et de la Forêt, Faculté de Foresterie, de Géographie et Géomatique, Université Laval, Québec, QC, G1V 0A6, Canada; State Key Laboratory of Systematic and Evolutionary Botany, Institute of Botany, Chinese Academy of Sciences, Beijing 100093, China; University of Chinese Academy of Sciences, Beijing 100049, China; National Engineering Research Center of Tree Breeding and Ecological Restoration, Beijing Advanced Innovation Center for Tree Breeding by Molecular Design, Key Laboratory of Genetics and Breeding in Forest Trees and Ornamental Plants, Ministry of Education, College of Biological Sciences and Technology, Beijing Forestry University, Beijing 100083, China

## Abstract

The genus *Rhododendron* (Ericaceae), with more than 1000 species highly diverse in flower color, is providing distinct ornamental values and a model system for flower color studies. Here, we investigated the divergence between two parental species with different flower color widely used for azalea breeding. Gapless genome assembly was generated for the yellow-flowered azalea, *Rhododendron molle*. Comparative genomics found recent proliferation of long terminal repeat retrotransposons (LTR-RTs), especially *Gypsy*, has resulted in a 125 Mb (19%) genome size increase in species-specific regions, and a significant amount of dispersed gene duplicates (13 402) and pseudogenes (17 437). Metabolomic assessment revealed that yellow flower coloration is attributed to the dynamic changes of carotenoids/flavonols biosynthesis and chlorophyll degradation. Time-ordered gene co-expression networks (TO-GCNs) and the comparison confirmed the metabolome and uncovered the specific gene regulatory changes underpinning the distinct flower pigmentation. B3 and ERF TFs were found dominating the gene regulation of carotenoids/flavonols characterized pigmentation in *R. molle*, while WRKY, ERF, WD40, C2H2, and NAC TFs collectively regulated the anthocyanins characterized pigmentation in the red-flowered *R simsii*. This study employed a multi-omics strategy in disentangling the complex divergence between two important azaleas and provided references for further functional genetics and molecular breeding.

## Introduction

As the largest genus of woody plants, *Rhododendron* comprises more than 1000 species widely distributed in natural settings throughout the temperate and montane tropical world. Among the diverse lineages of angiosperms, the genus is best-known for its great species diversity and magnificent flowers [1, 2]. Several high-quality *Rhododendron* genomes were released recently, including one draft genome [3] and eight pseudochromosome-level genomes [4–10], but no gapless genome has been obtained up to now. Gap-free or gapless genome assemblies have been accomplished in banana [11], rice [12], and Arabidopsis [13] with the developments of third-generation sequencing technologies. A high-quality genome will bring valuable opportunities for the investigation of more genomic variations, and make it easier to target suitable mutants and develop them for genetic and breeding research.

Transposable elements (TEs), the most difficult parts of ‘dark matter’ regions, are ubiquitous and abundant genome sequences that can adaptably move into new locations in the host genomes, multiply, and integrate there [14]. TEs can generate genetic novelty at the molecular level via both active and passive modes [15, 16] by causing *de novo* gene birth [17] and pseudogenization [18], or homology-driven ectopic (non-allelic) DNA recombination. TEs can also passively cause large-scale chromosomal rearrangements such as inversions and fusions [19, 20]. These features make TEs ideal facilitators of genotypic evolution [21]. However, our understanding of how TEs contribute to genetic variation is limited in azaleas, partly because TEs are difficult to accurately identify by short-read sequencing, because they are highly homologous and exist in large numbers.

In angiosperms, flower color is one of the most considerable and well-studied traits because it exhibits a bewildering diversity across evolutionary groups or individuals over a range of spatial, geographic, and temporal scales. Flower color shifts have occurred repeatedly in angiosperms, largely reflecting adaptation to novel pollinator regimes, thus facilitating speciation [22, 23]. Rhododendrons are valued for their marvelous range of flower colors, which are mainly determined by two major groups of pigments: flavonoids and carotenoids [5, 24–28]. Flower color shifts occurred repeatedly in the main clades of *Rhododendron* and even within species [25, 29], which was confirmed earlier by results of pigment analyses [25–28]. The complexity of the variance in flower colors appears to be comparable to the diversity of the genus’s numerous species, and the *Rhododendron* has the potential to serve as a model to probe the evolution of flower color. *Rhododendron molle* is a perennial shrub native to East Asia [10], and is the yellow-flowered parent of many cultivated hybrids. However, the biochemical and molecular basis for the petal color formation remains unclear, although the genome of a red-flowered parent species of widely cultivated azaleas was assembled and the entire metabolic pathways for flower pigmentation were reconstructed recently [5].

Here, we achieved a gapless genome assembly of *R. molle*, the highest quality assembly publicly available in the genus to date. More than 99% of the assembled genome were anchored on 13 chromosomes with seven in gap-free. Comparative genomic analyses enabled us to track the highly dynamic expansion of specific LTR-RT superfamilies in *R. molle*. We discovered that LTR-RT proliferations were important forces underpinning genomic divergence between *R. molle* and the red-flowered *R. simsii*. Furthermore, we unraveled the metabolic dynamic of the yellow-flowered azalea and investigated the remodulation of gene regulation during flower pigmentation through integrating metabolomic and time-ordered gene co-expression analyses, and compared with the red-flowered azalea. The gapless genome and the perception on the molecular mechanisms of flower coloration presented valuable resources for further genomic investigation and genetic breeding in azalea.

## Results

### Gapless genome assembly and annotation for *R. molle*

We produced approximately 51.97 giga bases (Gb) (100× coverages) of Oxford Nanopore Technologies (ONT) long sequencing reads, 49.12 Gb of PCR-free Illumina paired-end sequencing data, and 193.78 Gb of Hi-C sequencing data (Fig. S1; Tables S1 and S2, see online supplementary material). The final genome size was 653.46 mega bases (Mb), similar to the estimated genome size of 640 Mb based on 17-mer counting (Fig. S2, see online supplementary material). A telomere-to-telomere gapless genome assembly was obtained, with 13 pseudochromosomes anchoring ~99.48% of the assembled sequences (Table S3, see online supplementary material). The highly continuous gapless assembly was achieved with 34 contigs, with a contig N50 of 44.85 Mb, thus, with much longer sequence continuity than for all previously released genome assemblies in *Rhododendron* [3–6, 8]. Our assembly presented significant improvement (64 folds in contig N50) in continuity over an assembly recently reported for the same species [10], which is with 3764 contigs and contig N50 value of 0.71 Mb (Fig. S3; Table S1, see online supplementary material).

The assembly’s high fidelity was supported by two high mapping rates of 99.5% (ONT) and 99.6% (Illumina), two high 20-fold minimum genome coverages of 99.6% (ONT) and 95.4% (Illumina), and a single nucleotide polymorphism (SNP)-based heterozygosity level of ~0.52% after mapping the Illumina/ONT reads onto final assembly (Table S3, see online supplementary material). Finally, 93.4% of the complete Plantae BUSCO (Benchmarking Universal Single-Copy Orthologs) genes were identified (Table S3**,** see online supplementary material), indicative of the assembled genome’s high quality.

**Figure 1:**
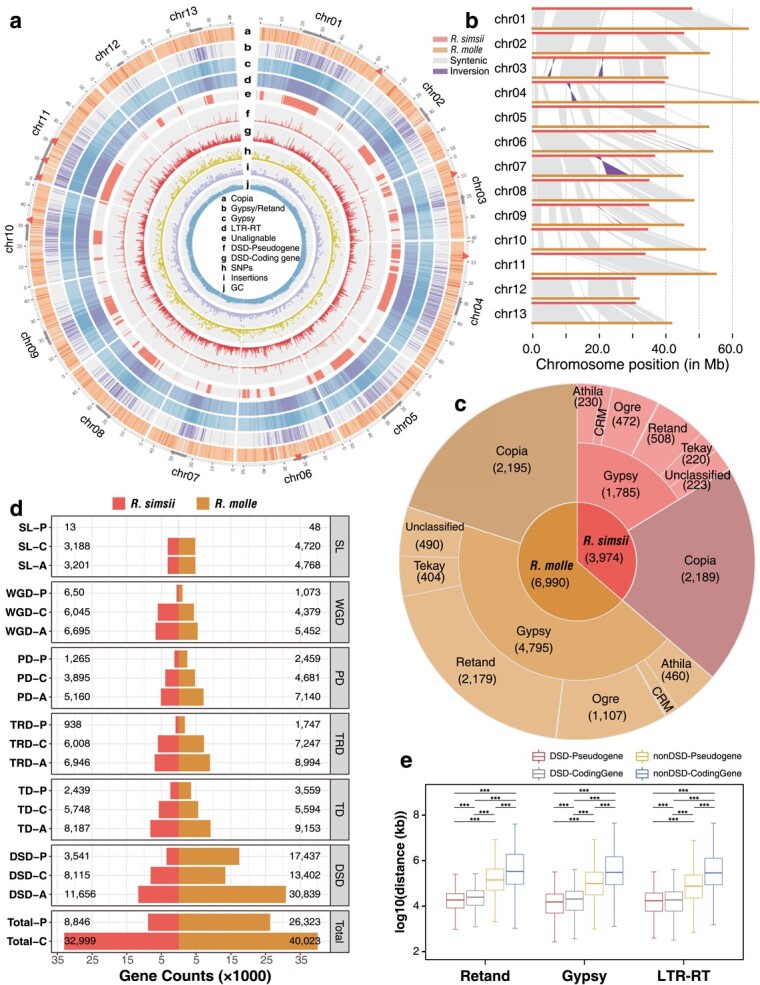
Genome comparison between *R. simsii* and *R. molle*. **a**, Synteny and distributions for genomic features of *R. molle* (**a**–**d**, the density of *Copia* LTR-RTs, Retand subfamily of *Gypsy* elements, *Gypsy* LTR-RTs, and LTR-RTs; **e**, unalignable regions between *R. simsii* and *R. molle*; **f**, pseudogene density; **g**, coding gene density; **h**, distribution of SNPs; **i**, distribution of insertions; **j**, histogram of GC contents). The genome assembly of *R. simsii* as the reference. The gray line segment in the outer circles indicates the potential pericentromeric region for each of the 13 chromosomes. The red triangle in the outer circles indicates gaps in assembly of *R. molle*. **b**, Structural variations between the reference *R. simsii* and *R. molle* genomes. *R. simsii* as the reference. **c**, Counts of all *Copia* and *Gypsy* LTR-RTs between *R. simsii* and *R. molle*. **d**, Barplot shows the counts of coding genes and pseudogenes from different modes of duplication between *R. simsii* and *R. molle*. SL-P: singleton pseudogenes; SL-C: singleton coding genes; SL-A: singleton all pseudogenes and coding genes. WGD, TD, PD, TRD and DSD respectively represent the five types of gene duplication: whole-genome, tandem, proximal, transposed, and dispersed duplications. **e**, Distributions of distances between different LTR-RTs and different gene sets of *R. molle*. In the boxplot representations, outliners are masked. nonDSD: other genes besides DSD duplicates. Mann–Whitney Wilcoxon test: ^***^P < 0.001.

One putative centromere was identified for each of the 13 pseudochromosomes, with length of the centromeres ranging from 3 Mb to 20 Mb (Fig. 1a; Fig. S4 and Table S4, see online supplementary material). We found that LTR retrotransposons, particularly *Gypsy* elements, were enriched preferentially in the centromeric regions identified, but not for *Copia* elements along the chromosomes (Fig. 1a). Guanine-Cytosine (GC) content was found to decreased and gene density increased from the centromeres toward the chromosome ends (Fig. 1a). By searching for occurrence of the telomeric characterized motif (TTTAGGG) along the chromosomes, 17 potential telomeric regions were revealed, among which a maximum of 951 and a minimum of 306 motif repeats were observed (Fig. S4 and Table S5, see online supplementary material). Two telomeres were identified at both ends of the six chromosomes (chr03, chr04, chr06, chr07, chr08, chr09), and one telomere was identified at one end of five chromosomes (chr01, chr02, chr10, chr12, chr13), respectively (Fig. S4 and Table S5**,** see online supplementary material). Seven chromosomes (chr02, chr05, chr07, chr08, chr09, chr12, chr13) presented as a single scaffold each, with chr07 and 08 being entirely reconstructed as gap-free and telomere to telomere (Fig. 1a; Fig. S4 and Table S6, see online supplementary material). The entire genome assembly showed only nine gaps localized on six chromosomes, including four gaps within extremely repetitive centromeric regions on chromosome chr11 (Fig. 1a; Fig. S5, see online supplementary material).

We predicted 41 600 genes from the *R. molle* genome based on 35.94 Gb RNA sequencing reads (Table S7, see online supplementary material). The average lengths of the total gene sequences, transcripts, coding sequences (CDSs), exon sequences, and intron sequences were calculated as 4492.4, 1466.6, 1118.7, 264.2, and 888.0 bp, respectively (Table S8, see online supplementary material). In all, 40 023 protein coding genes were predicted with a complete BUSCO recovery score of 93.7% (Table S9, see online supplementary material), which is in line with previous reports for azaleas (Table S1, see online supplementary material). In addition, we predicted 98 rRNAs, 477 tRNAs, and 1002 other non-coding RNAs (Table S8, see online supplementary material). A total of 36 585 (91.41%) protein-coding genes could be annotated against at least one of the databases for gene function, such as the Gene Ontology (GO) database (64.6%) (Table S10, see online supplementary material). The number of the predicted coding genes in the recently reported assembly of the same species was slightly lower (39 288 coding genes), and the proportion of functionally annotated genes was also lower (45.71% in GO database) (Table S1, see online supplementary material).

### Genome divergence and dispersed pseudogenization in azaleas

While significant interspecific collinearity occurred at the gene level (Fig. S6, see online supplementary material), whole genome alignment revealed structural and local genomic divergence between the genomes of *R. simsii* and *R. molle* (Fig. 1b). The latter genome is 23.6% larger (653.46 Mb) than that of *R. simsii* (528.64 Mb). A total of 56 structural rearrangements were found, 27 inversions (12 853 461 bp), 20 translocations (91 021 bp), and nine duplications (44 318 bp) (Fig. S7, see online supplementary material). Among the local sequence differences, 138 751 SNPs, 12 885 insertions, and 12 344 deletions were identified (Fig. 1a; Fig. S7, see online supplementary material). In addition to the 402 253 777 bp long syntenic regions (~60% of the entire *R. molle* genome), 234 864 123 bp (~36%) were specific to *R. molle* and thus unaligned to the reference genome (Fig. 1b; Fig. S7, see online supplementary material). These unalignable regions were LTR-RT enriched regions within the *R. molle* genome (a–e in Fig. 1a), suggesting that LTR-RTs likely drove the interspecific genomic divergence. Interestingly, we found most of the unique sequences (regions unalignable to *R. simsii*) of *R. molle* are of the potential centromeric regions (Fig. 1a), indicating that the difference of centromere between these two species and the recent formation of centromeres contributed to species divergence.

We found that the *Gypsy* superfamily dominated the expansion of genome size within *R. molle*. We predicted 668 841 repeat elements (349 487 787 bp; 53.48%) in the *R. molle* genome, among which 42.41% LTR-RTs, 4.99% uncharacterized TEs, and 2.70% DNA transposons (Table S11, see online supplementary material). In fact, the *Gypsy* superfamily represented the highest portion (27.86% of the *R. molle* genome) among LTR-RTs (Table S11, see online supplementary material), and far more *Gypsy* sequences than in *R. simsii* (11.90% of its genome sequence). LTR-RTs in *R. molle* were classified into 25 954 solo-LTR-RTs (S), 3751 truncated (T), and 3239 intact (I) LTR-RTs (Fig. S8 and Table S12, see online supplementary material). The intact and truncated LTR-RTs were primarily classified to 4795 *Gypsy* and 2195 *Copia* elements (Fig. 1c). The expansion of *Gypsy* in *R. molle* compared to *R. simsii* was mainly attributed to the expansion of four subfamilies: Retand (2179), Ogre (1107), Athila (460), and Tekay (404) (Fig. 1c; Table S12, see online supplementary material). Similar to *R. simsii*, most intact LTR-RTs across the *R. molle* genome showed a burst of their accumulation within recent 2 Mya (Fig. S9, see online supplementary material). *R. molle* also featured a higher LTR-RTs accumulation (S + T + I = 32 944) and a high removal rate (proportions of LTR clusters with S:I > 3) (Table S13, see online supplementary material).

LTR-RT and *Gypsy* expansions were also associated with a remarkable number of gene duplications and pseudogenization. For *R. molle* we identified 26 323 pseudogenes and 40 023 coding genes, which represent much higher numbers than those in *R. simsii* (pseudogenes: 8846; coding genes 32 999) (Fig. 1d). Among coding genes and pseudogenes in *R. molle*, dispersed duplicates (DSD) were the most abundant with 30 839 (46.5%), followed by 9153 tandem duplicates (TD; 13.8%), 8994 transposed duplicates (TRD; 13.6%), 7140 proximal duplicates (PD; 10.8%), and 5452 whole-genome duplicates (WGD; 8.2%) (Fig. 1d; Fig. S10, see online supplementary material). The recent DSD burst was the most frequent entity both for DSD-coding genes (13402) and DSD-pseudogenes (17437) (Fig. 1d; Fig. S10, see online supplementary material). In *R. molle*, we found 5287 more DSD-coding genes and 13 896 more DSD-pseudogenes than in *R. simsii*. It should be noted that DSD-coding genes and DSD-pseudogenes were found significantly closer to LTR-RTs, especially to the Retand subfamily of *Gypsy* superfamily which was the dominant one (f, g in Fig. 1a and 1e).

### Metabolome and pigment biosynthesis

**Figure 2:**
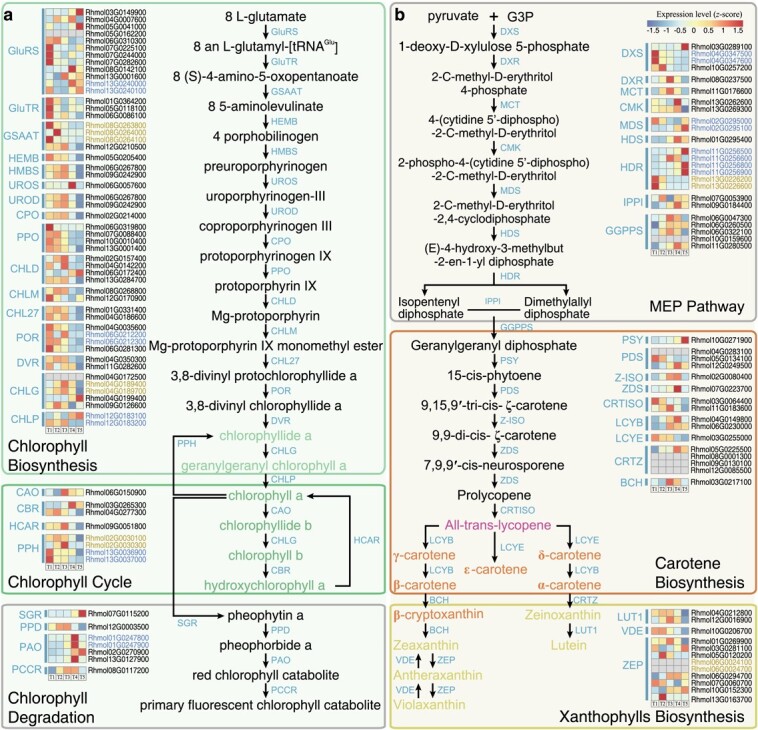
The metabolic pathways and time-ordered gene regulations of carotenoid and chlorophyll in *R. molle*. Genes located in proximal and tandem clusters are displayed as brown and blue, respectively. The heatmaps indicate the gene expression values at flowering time points (labeled T1-T5). The grey bars indicate the zero of the TPM. **a**, Chlorophyll metabolic pathways. The chlorophyll enzymatic genes divided to three groups: ‘Chlorophyll Biosynthesis’, ‘Chlorophyll Cycle’ and ‘Chlorophyll Degradation’, respectively. Genes encode the following enzymes: CAO: chlorophyllide-a: oxygen 7-oxidoreductase; CBR: chlorophyll b reductase; CHLD: magnesium chelatase; CHLG: chlorophyll synthetase; CHLM: magnesium protopophyrin IX methyltransferase; CHL27: magnesium-portoporphyrin IX 13 -monomethyl ester cyclase; CHLP: geranylgeranyl-chlorophyll a reductase; CPO: coproporphyrinogen oxidase; GluRS: glutamyl-tRNA synthetase; DVR: 3,8-divinyl-chlorophyllide 8-vinyl reductase; GluTR: glutamyl-tRNA reductase; GSAAT: glutamate-1-semialdehyde 2,1-aminomutase; HCAR: 7-hydroxychlorophyll a reductase; HEMB: porphobilinogen synthase; HMBS: hydroxymethylbilane synthase; PAO: Pheophorbide a oxygenase; PCCR: red chlorophyll catabolite reductase; POR: light-independent 3,8-divinyl-protochlorophyllide reductase; PPD: pheophorbidase; PPH: pheophytinase; PPO: protoporphyrinogen oxidase; SGR: magnesium dechelatase; UROD: uroporphyrinogen decarboxylase; UROS: uroporphyrinogen III synthase. **b**, Carotenoid metabolic pathways. The carotenoid enzymatic genes divided into three groups: ‘2-C-methyl-D-erythritol 4-phosphate (MEP) Pathway’, ‘Carotene Biosynthesis’ and ‘Xanthophylls Biosynthesis’, respectively. Genes encode the following enzymes: BCH: Beta-carotene 3-hydroxylase; CMK: 4-(Cytidine 5′-diphospho)-2-C-methyl-D-erythritol kinase; CRTISO: Prolycopene isomerase; CRTZ: beta-ring hydroxylase; DXS: 1-deoxy-D-xylulose-5-phosphate synthase; DXR: 1-Deoxy-D-xylulose-5-phosphate reductoisomerase; GGPPS: Geranylgeranyl diphosphate synthase; HDR: 4-Hydroxy-3-methylbut-2-enyl diphosphate reductase; HDS: 4-Hydroxy-3-methylbut-2-enyl-diphosphate synthase; IPPI: isopentenyl-pyrophosphate isomerase; LCYB: Lycopene beta-cyclase; LCYE: Lycopene epsilon-cyclase; LUT1: Carotene epsilon-hydroxylase; MCT: 2-C-methyl-D-erythritol 4-phosphate cytidylyltransferase; MDS: 2-C-methyl-D-erythritol 2,4-cyclodiphosphate synthase; PDS: 15-cis-phytoene desaturase; PSY: 15-cis-phytoene synthase; VDE: Violaxanthin de-epoxidase; ZDS: 9,9′-di-cis-zeta-carotene desaturase; ZEP: Zeaxanthin epoxidase; Z-ISO: Zeta-carotene isomerase.

**Figure 3:**
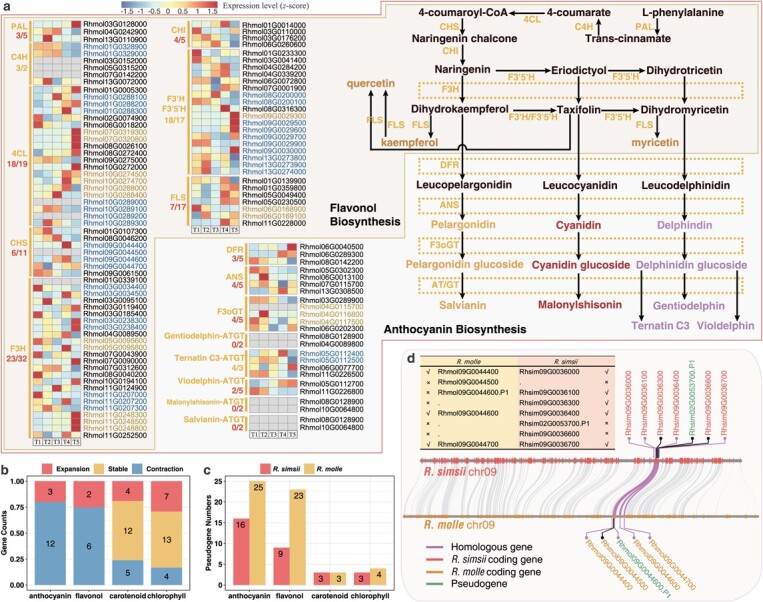
Loss of anthocyanin biosynthetic genes leads to lack of red varieties in *R. molle*. **a**, The metabolic pathways and time-ordered gene regulations of the two types of flavonoids: flavonols and anthocyanins. Genes located in proximal and tandem clusters are displayed as brown and blue, respectively. The heatmaps indicate the gene expression values (in normalized TPMs) at flowering time points (labeled T1–T5). The grey bars indicate the zero of the TPM. All genes are divided into two groups: ‘flavonol biosynthesis’ and ‘anthocyanin biosynthesis’. The fractions below the gene abbreviation indicate the ratio of the number of expressed genes (average TPM > 0) from the *R. molle* and *R. simsii*; the numerator is the gene number of *R. molle* and the denominator is the gene number of *R. simsii*. An enzymatic gene family is expanded if the number of genes of *R. molle* is greater than that of *R. simsii*, e.g. C4H family (3:2). Conversely, the family is a contracted family, such as PAL family (3:5). Genes encode the following enzymes: 4CL: 4-coumarate CoA ligase; ANS: anthocyanidin synthase; AT: acyltransferase; C4H: cinnamate-4-hydroxylase; CHI: chalcone isomerase; CHS: chalcone synthase; DFR: dihydroflavonol reductase; F3H: flavanone 3-hydroxylase; F3’H: flavonoid 3′-hydroxylase; F3’5’H: flavonoid 3′,5′-hydroxylase; F3oGT: flavonol-3-O-glucosyl transferase; FLS: flavonol synthase; GT: glucosyltransferase; PAL: phenylalanine ammonia-lyase. **b**, Barplot shows the counts of enzyme families from different metabolic pathways of anthocyanin, flavonol, carotenoid, and chlorophyll. If the fraction of gene numbers encoding one enzyme >1, then this enzyme is defined as a ‘expansion’ enzyme; the fraction = 1, then this enzyme is defined as a ‘stable’ enzyme; and the fraction <1, then this enzyme is defined as a ‘contraction’ enzyme. **c**, The pseudogene numbers from different metabolic pathways of anthocyanin, flavonol, carotenoid, and chlorophyll. We determine that a pseudogene encodes an enzyme based on what enzyme its parental coding gene encodes. **d**, Synteny patterns between *R. simsii* and *R. molle*. The patterns illustrate gene gain/loss after gene duplication within an ancestral region. These collinear relationships are highlighted by purple lines. In the list of genes in the upper left corner, a check mark indicates that the gene retains its ancient function, a x mark indicates loss of original function in various forms (pseudogenization, and gene silencing), and a dot indicates that the opposite gene was deleted or formed after diversification of *R. simsii* and *R. molle*.

We reconstructed the pigment metabolic pathways (chlorophylls, carotenoids, anthocyanins, and flavonols) potentially linked to flower color formation of *R. molle* and *R. simsii* (Figs. 2 and 3a; Figs. S11 and Figure S12, see online supplementary material). Consistent with findings in the red flowering *R. simsii* (55 genes; 44%), in the yellow flowering *R. molle* more enzymatic genes were present in tandem (TD) or proximal (PD) duplication (47 genes; 45.19%) within the anthocyanin metabolic pathway as compared to the chlorophyll/carotenoid biosynthesis pathways (25% on average) (Table S14, see online supplementary material). Enzymatic genes of the anthocyanin/flavonol metabolic pathway showed a higher proportion of PD (15.31% versus 7.91%), while a lower proportion of TD (28.82% versus 35.25%) in *R. molle* than in *R. simsii*.

We compared the sizes of gene families between *R. molle* and *R. simsii* and found family size contractions in 12 out of 15 families in the anthocyanin biosynthesis pathway, and its degree of contraction (80%) was highest among all biosynthetic pathways examined in *R. molle* (Fig. 3b; Table S15, see online supplementary material). The largest number of pseudogenes was also identified in gene families of anthocyanin synthesis in *R. molle*, suggesting that pseudogenization may have contributed to the observed gene family contractions in this species (Fig. 3c). By comparison, in both chlorophyll and carotenoid metabolic pathways, contracted families and pseudogenes were relatively rare (Fig. 3b and c). Because flavonols share seven biosynthetic steps with anthocyanins (Fig. 3a), a considerable number of pseudogenes and contracted families was also found in the flavonol pathway (Fig. 3b and c).

Based on conserved sequence motifs and domains, Chalcone synthase (CHS) proteins, the first committed enzyme in the flavonoid biosynthesis, could be divided into C1–1/2 clades (group I) and a C2 clade (group II) (Fig. S13, see online supplementary material). Group II (PLN03173) showed significant gene gain in *R. simsii* while gene loss in *R. molle* (Fig. S13, see online supplementary material). The interspecific syntenic block of the C1–1 clade provides a good example for how gene gain and loss occurred (Fig. 3d). It was discovered that gene duplications significantly conduced to the expansions of gene families by causing duplications (e.g. tandem duplicates: Rhmol09G0044400 and Rhmol09G0044500). Gene loss was found driven by pseudogenization (Rhmol09G0044600.P1), with the potential loss of the original function through either gene silencing (Rhmol09G0044500) or gene deletion of homologous genes (Rhsim09G0036300).

**Figure 4:**
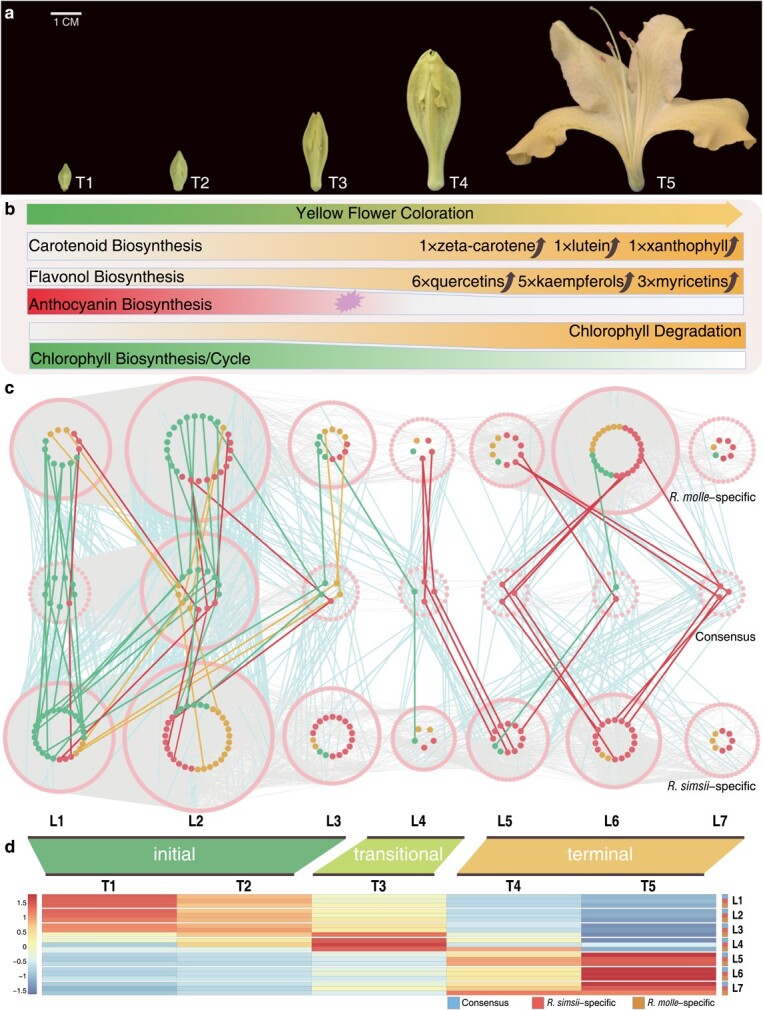
Pigmentation changes and time-ordered gene co-expression networks related to yellow flower coloration. **a**, Five flower developmental time points (T1–T5). **b**, Pattern of pigmentation changes during yellow flower coloration of *R. molle* in metabonomics between T5 vs T3. **c**, Predicted gene co-expression networks and the connections among TFs and enzymatic genes involved in biosynthetic pathways of carotenoids (orange points), chlorophylls (green points), and anthocyanins/flavonols (red points). Pink nodes represent TFs. L1 to L7 indicate the levels identified in three f-ordered time-ordered gene co-expression networks (*R. molle*-specific, *R. simsii*-specific, and the consensus TO-GCNs). Edges were not shown between enzymatic genes. **d**, Heatmaps of average TPMs (z-score normalized) at each level of TO-GCNs at each flowering time point. Three flower coloring stages were identified as the initial (T1-T2), transitional (T3), and terminal (T4-T5) stages, based on the expression profiles.

Corolla samples from five different developmental stages (T1 to T5) were used for transcriptomic and metabolomic analyses (Fig. 4a; Fig. S14, see online supplementary material). To confirm the pigments determining petal coloration in *R. molle*, we conducted metabolomic assessments between two representative time points T3 and T5, which are tightly associated with color shift from green to yellow (Fig. 4b). A total of 10 734 mass features were identified and 6637 compounds were annotated and used for data interpretation (Fig. S15, see online supplementary material). A higher number of carotenoids/flavonols was upregulated at T5 (Fig. S16, see online supplementary material). The upregulated flavonol metabolites included six quercetins, five kaempferols, and three myricetins. We noticed a significant accumulation of zeta-carotene, lutein, and xanthophyll in the final stage (T5) (Fig. 4b). In sum, yellow flowering may have been characterized and driven by carotenoids/flavonols biosynthesis, chlorophyll degradation, but not the anthocyanin biosynthesis, which is consistent with the gene loss uncovered in the specific metabolic pathways.

### Comparative analyses of time-ordered gene co-expression between azaleas

We constructed the time-ordered gene co-expression networks (TO-GCNs) separately for two azaleas, and then compared these two networks to investigate the gene regulation associated with flower color shift between two azaleas. Two species-specific TO-GCNs (*R. molle*-, *R. simsii*-specific) as well as a consensus TO-GCN were isolated (Fig. 4c). Seven time-ordered subnetworks (here levels: L1-L7) were reconstructed for each TO-GCN and further examined (Fig. 4c). We identified 950 (817 transcription factor (TF) and 133 enzymatic), 895 (736 and 159), and 372 (323 and 49) genes, which are related to the carotenoid/anthocyanin/flavonoid/chlorophyll biosynthetic pathways, in the *R. molle*-specific, *R. simsii*-specific, and consensus TO-GCNs, respectively (Table S16**,** see online supplementary material). Based on expression patterns during flower pigmentation, the time-ordered subnetworks could be summarized into three major stages: initial (T1-T2; corresponding to L1-L3, when petals were still green), transitional (T3; corresponding to L4, during green-to-yellow flower transition), and terminal (T4-T5; corresponding to L5-L7, with yellow color) (Fig. 4d).

**Figure 5:**
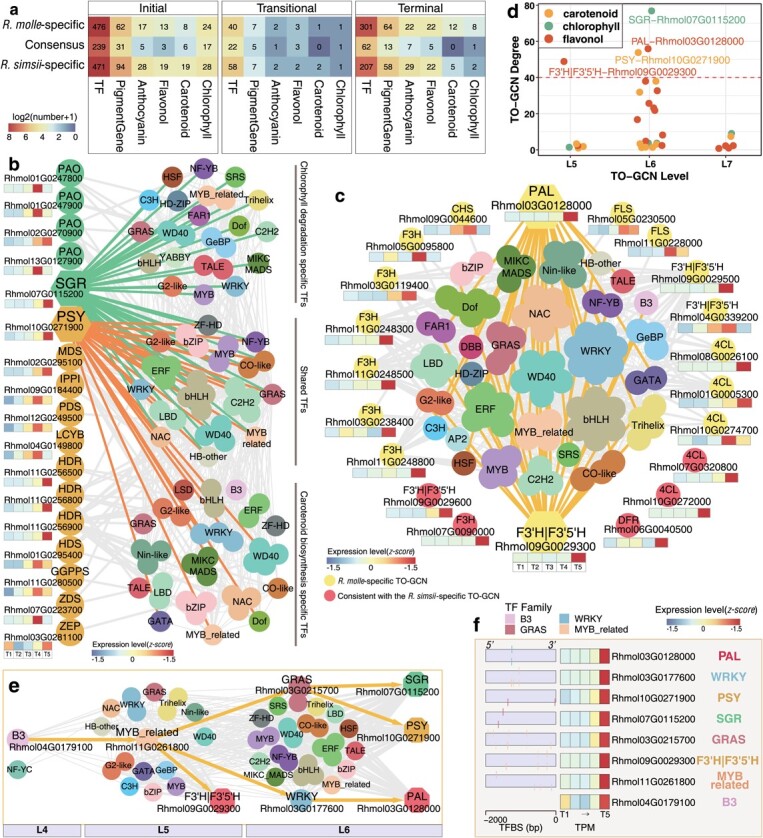
Subnetworks for pigmentation metabolic pathways at the terminal stage of the *R. molle*-specific TO-GCN. **a**, Numbers of TFs and enzymatic genes involved in metabolic pathways for carotenoids, chlorophylls, and anthocyanins/flavonols at three stages (the initial, transitional, and terminal stages) among three TO-GCNs (*R. molle*-specific, *R. simsii*-specific, and the consensus TO-GCNs). **b**, Subnetwork of the *R. molle*-specific TO-GCN for carotenoid biosynthesis and chlorophyll degradation. **c**, Subnetwork of the *R. molle*-specific TO-GCN for flavonol biosynthesis. Edges were not shown between TFs and five enzymatic genes (red points), which were consistent with the *R. simsii*-specific TO-GCN. **d**, Degree of 34 enzymatic genes of the chlorophyll degradation and carotenoid/flavonol biosynthesis in two subnetworks as shown in **a** and **b**. The degree cutoff for hub gene is 40. **e**, Resolved hierarchical regulations for four hub genes *PSY*, *SGR*, *PAL,* and *F3’H|F3’5’H*. **f**, Gene expressions (TPM) and TF binding site (TFBS) detected in the 2 Kb upstream sequences of four hub genes and four potential regulators (here, TFs; *GRAS, WRKY, MYB_related*, and *B3*) in *R. molle.* Edges are not shown between enzymatic genes.

The most significant difference between the two azaleas was seen at the terminal stage, when examining the number of species-specific genes. A wide variety of genes showed conserved co-expression (consensus) between two azaleas at the initial stage, including 239 TFs and 31 enzymatic genes associated with pigment biosynthesis (Fig. 5a). At the initial stage, chlorophyll biosynthetic genes were the most abundant type (17) among the enzymatic genes, followed by carotenoid biosynthetic genes (6), anthocyanin biosynthetic genes (5), and flavonol biosynthetic genes (3). At the terminal stage, the limited consistency of TO-GCNs between the two azaleas (Fig. 5a) facilitated the identification of species-specific pigment regulation. Genes of carotenoids/flavonols biosynthesis were found characterized in the subnetworks of the yellow-flowered *R. molle*, while anthocyanin biosynthetic genes were recognized in the subnetwork of the red-flowered *R simsii*, further confirming the metabolome assessment, at the terminal stage.

Key regulatory and enzymatic genes and their hierarchical regulation were predicted by examining the species-specific networks. In the two *R. molle*-specific subnetworks, a total of 33 enzymatic genes from chlorophyll degradation (five genes), carotenoid biosynthesis (12 genes) (Fig. 5b), and flavonol biosynthesis (16 genes) (Fig. 5c) were found dominated with high expression levels at the terminal stage. Among the 90 TFs that appeared as the possible regulators of these enzymatic genes, none of the family was found dominant in the subnetwork associated with chlorophyll and carotenoid (Fig. 5b). In the subnetwork for flavonol biosynthetic genes (Fig. 5c), members of five TF families were identified as the potential direct regulators, and these TFs are of WRKYs (eight genes), basic helixloop-helix (bHLHs) (seven), WD40s (six), NACs (six), and ethylene-responsive element binding factors (ERFs) (six). We further identified hub genes of *SGR* (Rhmol07G0115200), *PSY* (Rhmol10G0271900), *PAL* (Rhmol03G0128000), and *F3’H|F3’5’H* (Rhmol09G0029300) families (Fig. 5d), which all represent important enzymes catalyzing chlorophyll degradation and carotenoid/flavonol biosynthesis producing yellow pigments. This finding was again in line with our metabolome assessment. By examining the network topology, we could predict that these four hub genes might be regulated in the hierarchical orders with two TFs as the third regulators, either 17 TF genes as the intermediate second regulators, or 27 TFs as the direct regulators (Fig. 5e). The TF binding site (TFBS) predictions for *R. molle* further confirmed that *PSY/SGR* genes may be co-regulated by *GRAS* (Rhmol03G0215700), *MYB_related* (Rhmol11G0261800), and *B3* (Rhmol04G0179100) in a hierarchical manner. Further, the B3 and MYB_related TFs were recognized as the key upstream regulators regulating *WRKY* (Rhmol03G0177600), *F3’H|F3’5’H* (Rhmol09G0029300) and *PAL* (Rhmol03G0128000) in a hierarchical manner (Fig. 5f).

**Figure 6:**
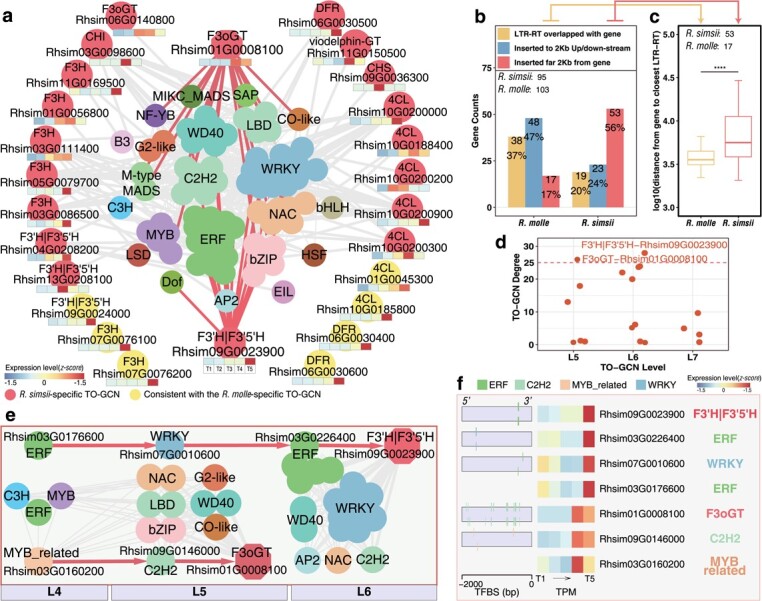
Subnetwork for pigmentation metabolic pathways at the terminal stage of the *R. simsii*-specific TO-GCN. **a**, Subnetwork of the *R. simsii*-specific TO-GCN for anthocyanin biosynthesis. Edges were not shown between TFs and seven enzymatic genes (yellow points), which were consistent with the *R. molle*-specific TO-GCN. **b**, The three bars on the left indicate the counts of 95 genes were chosen at the terminal stage in the *R. simsii*-specific TO-GCN after shielding consensus genes with the *R. molle*-specific TO-GCN as shown in **a**. Then the three bars on the right indicate the counts of 103 potential orthologs in *R. molle*. The yellow bar indicates LTR-RTs overlapped with the genes, blue bar indicates the LTR-RTs inserted into 2 kb upstream or downstream of the genes, and red bar indicates the LTR-RTs inserted out of 2 kb upstream or downstream of the genes. **c**, Distributions of distances between closest LTR-RTs and genes far 2 Kb from LTR-RTs of *R. molle* and *R. simsii*. In the boxplot representations, outliners are masked. Student’s *t* test. ^***^P < 0.001. **d**, Degree of 19 enzymatic genes of the anthocyanin biosynthesis in the subnetwork as shown in **a**. The degree cutoff for hub gene is 25. **e**, resolved hierarchical regulation for two hub genes *F3oGT* and *F3’H|F3’5’H*. **f**, Gene expressions (TPM) and TF binding sites (TFBS) detected in the 2 Kb upstream sequences of two hub genes and five potential regulatory elements (here, TFs; *ERF*, *WRKY*, *C2H2*, and *MYB_related*) in *R. simsii*. Edges are not shown between enzymatic genes.

In the *R. simsii*-specific subnetwork, we identified 19 dominant enzymatic genes which are from the red pigment pathways producing anthocyanin, and all these genes were found expressed with high levels at the terminal stage (Fig. 6a). By examining the hierarchical network, we could predicate these enzymatic genes might be regulated by 74 potential regulators, mostly WRKY (13 genes), ERF (13), WD40 (seven), C2H2 (seven), and NAC (six) family members. Interestingly, the 103 putative orthologs of the 95 genes dominated in the *R. simsii*-specific subnetwork were significantly associated with the drastic LTR-RT insertions in *R. molle* (Fig. 6b, c). We further detected the hub genes *F3oGT* (Rhsim01G0008100) and *F3’H|F3’5’H* (Rhsim09G0023900) (Fig. 6d) and 35 of their potential regulators (five within L4; 12 in L5, and 18 in L6) acting in a hierarchical order (Fig. 6e). Two hierarchical gene expression regulatory networks were supported by the TFBS prediction for *R. simsii*, and these gene regulations were: (i) *F3’H|F3’5’H* may be co-regulated by ERF (Rhsim03G0226400), WRKY (Rhsim07G0010600), and ERF (Rhsim03G0176600); and (ii) *F3oGT* may be co-regulated by C2H2 (Rhsim09G0146000) and MYB_related (Rhsim03G0160200) (Fig. 6f).

## Discussion

We generated the first gapless genome assembly in azaleas with the combination of long-read sequencing and Hi-C scaffolding technologies. The contig N50 of our assembly is 44.9 Mb, which is significantly higher than that of any other genome assemblies available for azaleas [3–10]. The highest proportion (99.4%) of the genome has been anchored on 13 chromosomes with the least nine gaps in our genome assembly. The gapless genome assembly presents unique values for further genomic and functional investigation, and molecular breeding, such as genome editing in azaleas.

TEs are powerful facilitators of evolutionary change at both genomic and genetic levels [19, 30]. Our comparative genomic results indicated that dynamic changes in LTR-RTs can lead to genome size increase as well as the generation of large amounts of non-collinear species-specific regions. We saw further evidence for LTR-RT driven expansion of duplicate genes, particularly as dispersed duplications, and a distinct amount of pseudogenization resulting from LTR-RT insertions, especially from the *Gypsy* superfamily. Comparative genomics and transcriptome profiling in the present study unraveled LTR-RTs may have facilitated gene loss and in turn remodeled and disrupted anthocyanin biosynthesis, in *R. molle*. Our results are in line with previously conducted research on TEs that were found to have mediated gene duplication as well as restructured genes and entire genomes [31, 32]. Our study revealed a clear pathway of TE-induced genome divergence. Structural and genetic innovations are usually considered as active or passive products of the activity of LTR-RTs, which are ubiquitous and widespread [15]. While these features demonstrate the enormous potential of LTR-RTs in generating variants, they also pose a major challenge to accurately explore the specific functions of LTR-RTs [21]. Therefore, choosing an appropriate entry point into their exploration represents a critical factor.

Here, we defined the putative centromeric regions for each chromosome based on profile of Hi-C chromatin contact, which could be used for additional verification of centromere regions by procedures like ChIP-seq with an antibody against the quickly evolving CENH3 protein. Further, we found the high-density *Gypsy* retrotransposons, especially elements from Retand subfamily, were enriched in the identified centromere regions, and most of the centromere regions of *R. molle* are of unique sequences unalignable to *R. simsii*. Retrotransposons are widespread in the centromeres of diverse plants and can directly contribute to centromere formation and evolution [33, 34]. The unique sequence composition of centromeres in *R. molle* was indicated that the centromeres are not conserved between *R. simsii* and *R. molle*, which may further indicate the expanded *Gypsy* elements may contribute much to divergent evolution and formation of the species-specific centromeres in azalea. Centromere sequences are conserved within species but divergent between species, which has been observed for model taxa [35]. We identified and characterized possible centromeric regions in azaleas for the first time. The high-continuity gapless assembly allowed us to explore the ‘dark matter’ regions on the azalea genome, such as telomeres, centromeres, and highly repetitive elements.

Flower color is the hallmark of angiosperm diversity, as changes in flower color have led to major evolutionary innovations by attracting different groups of pollinators [22, 23]. Flower coloration depends largely on the type and amount of pigments accumulating in petals; however, the genetics and biochemistry of floral pigments in angiosperms is poorly understood [5, 24–28], with the exception of some classical model plants [36, 37]. Here, with metabolome assessment, we revealed that the yellow flower of *Rhododendron* can be attributed to (i) synthesis of carotenoid and flavonol; (ii) chlorophyll degradation; and (iii) absence of anthocyanin synthesis. The entire metabolic pathways were reconstructed for carotenoids/chlorophyll/flavonol/anthocyanin in *R. molle*. Then, comparative analyses indicated that gene losses were common in anthocyanin biosynthetic gene families, and might lead to the lack of red pigment biosynthesis in *R. molle*. As a pervasive source of genetic variation, gene loss can be an adaptive evolutionary force that is associated with gene functions involved in phenotypic variation, such as flower color shift, when organisms are faced with abrupt environmental challenges [38, 39].

Previously, we described the time-ordered gene co-expression networks (TO-GCNs) for flower coloration in *R. simsii*, and inferred the potential regulatory relationships between transcription factors and pigment biosynthetic genes [5]. In this study, the comparative analyses of TO-GCNs revealed the consensus and specific hierarchical networks of yellow and red flowered azaleas. The *R. simsii*-specific TO-GCNs unraveled that the WRKY, ERF, WD40, C2H2, and NAC transcription factors (TFs) collectively regulate anthocyanins biosynthesis for the terminal stage of flower development. While the result was supported by previous reports [5], it also identified the more precise potential regulators in the *R. simsii*-specific TO-GCNs by considering the regulatory relationships in the consensus TO-GCNs. Previous reports on yellow-flowered azaleas did not provide much information on flower coloration [25, 40] and yellow flower colors have been the dream of azalea breeders. Our study uncovered that the B3 and MYB-related TFs were the major regulatory elements of the key biosynthetic genes controlling carotenoid/flavonol/chlorophyll metabolism in *R. molle*-specific TO-GCNs. The enzymatic genes and potential regulators of the related pigments biosynthetic pathways represent worthy priorities to further functional research during successive flower development stages in azaleas.

Here, we presented the first gapless genome assembly for azalea, and demonstrated the utility of such a multi-omics strategy for investigation of divergence between azaleas with distinct flower color, which also exhibit complex mechanisms, and thus serve as valuable resources for functional genetic studies and breeding in *Rhododendron*. Overall, the genomic resources will help researchers identify genetic and functional indicators and translate study results into genetic improvements for azalea horticultural research.

## Materials and methods

### Plant sampling, library preparation, and sequencing

For whole-genome assembly, a 10-year-old adult *R. molle* individual was selected from a natural population in Zhuzhou, Hunan Province, China. Fresh leaves were collected for whole genome sequencing with different platforms: Illumina HiSeq X Ten and Oxford Nanopore Technologies [ONT] sequencing. For RNA sequencing in support of gene annotation, young leaves, petal lower lips, stamens, young stems, and pistils were sampled from the same individual.

In order to identify transcriptomic and chemical changes during flower coloration, we collected corolla tissues from five trees with five flowering time points based on external characteristics. Twenty-five samples of corollas (five replicates per flowering time points) from these five time points were collected for transcriptomic sequencing (Table S17, see online supplementary material). In addition, we collected ten corolla samples (five replicates per stage) for a comparative metabolomic experiment: stage three vs. five (T3 vs. T5, the key conversion stages of color from green to yellow). All fresh tissues were harvested and immediately frozen in liquid nitrogen.

We extracted and purified the total DNA from fresh leaves. For the PromethION ONT platform, libraries with inserts of 20–40 kb were prepared and sequenced in a single cell. For Illumina short-reads sequencing, PCR-free libraries with 150-bp paired-end (PE) insert were prepared and sequenced on the Illumina HiSeq X Ten platform. For Hi-C sequencing, young and fresh leaf tissues were preserved in 1% (vol/vol) formaldehyde, DNA was cross-linked according to protocol, and a single library (150-bp PE) was amplified sequenced on the Illumina HiSeq X platform. More detailed information on sequencing can be seen in Note S1 (see online supplementary material).

### Genome assembly and assessment

The potential genome size was estimated with *K*-mer analysis (*K* = 17) by jellyfish [41]. Primary assembly were generated by two steps: NextDenovo v2.3.0 (https://github.com/Nextomics/NextDenovo) was used for correcting the ONT long-reads, followed by one round of polish conducted with Pilon (http://github.com/broadinstitute/pilon). The Hi-C data was mapped against the primary assembly to obtain normalized contact matrix with Juicer v1.7.6 (https://github.com/aidenlab/juicer). The Hi-C contact matrix was used for scaffolding with the 3D-DNA pipeline. Afterwards, we obtained the new scaffolds with gap filled with two iterations by LR_Gapcloser v1.0 (https://github.com/CAFS-bioinformatics/LR_Gapcloser) and five rounds of polishing by Nextpolish v1.2.4 [42]. After removing the redundancy of unanchored contigs using Redundans v0.14a (identity >0.98) [43], contigs were also removed which had less than half of the mean covering depth of the main peak (Table S18, see online supplementary material).

BUSCO [44] was implemented for assessing the completeness and continuity of genome assembly. For estimating the consensus error and mapping rates, the preprocessed reads from ONT, Illumina, RNA-seq and Hi-C data were mapped to the final assembly with bwa v0.7 (https://github.com/lh3/bwa), minimap2 v2.17 [45], HiSat2 v2 (https://github.com/infphilo/hisat2). For more details on genome assembly and quality control see Note S2 (see online supplementary material).

### Centromere and telomere identification

In the three-dimensional (3D) genomic architectures, the strong looping interactions were observed among sequences within the same centromere [46], and between sequences of different centromeres in the Rabl organization [47]. Based on the evidence, we mapped the corrected Hi-C sequencing data against the genome assembly and further identified the potential centromeres by Centurion [48]. For each chromosome, the centromere position was identified to a genomic point with one base pair in Centurion. With the Hi-C contact map, we further examined chromatin contacts and called the potential long-range centromeric regions manually.

As one of the basic nucleoprotein structures, Telomeres are usually located at the v ends of chromosomes of eukaryote genomes. In most plants, telomeres sequences are generally conserved as the tandemly arranged minisatellites with the formula of (TTTAGGG)n [49]. Here, telomeric regions were identified with the characteristic motif (TTTAGGG) in the current study. More details are available in Note S2 (see online supplementary material).

### Annotation of repeat elements and genes

Within the genome sequence of *R. molle*, repeat elements were identified *de novo* with RepeatModeler v1 (http://www.repeatmasker.org), and then the resulted annotations were used as a repeat library for a further round of annotation. Another intact LTR-RTs library was generated with LTR_retriever [50] and combined with the former *de novo* repeats library to construct a consensus repeats library, which was further used for annotating homologous-based repeats by RepeatMasker v4.0.5 (http://www.repeatmasker.org). The repeat elements for *R.simsii* were annotated with the same pipeline and parameters in our previous reports [5]. Furthermore, we investigated the annotation and age determination of LTR-RTs within both genomes of *R. molle* and *R. simsii* following a same and standard pipeline [51].

We predicted the protein-coding gene structures with the combination strategy of ab initio, transcriptome and homology-based gene prediction by MAKER2 pipeline [52]. For transcriptome-based prediction, *de novo* and referenced genome-based transcripts were constructed by HiSat2 v2.1.0 and Trinity [53]. All transcripts were then aligned to reference assembly with PASA [54] to predict the gene models. For the homology-based prediction, we used 187 124 nonredundant amino acid sequences from *Rhododendron delavayi* [3], *R. simsii* [5], *R. williamsianum* [4], *Vaccinium corymbosum* [55], *Actinidia chinensis* [56], and *Arabidopsis thaliana* [57] as homologous protein evidence. For the ab initio prediction, Augustus v3.2.3 (http://bioinf.uni-greifswald.de/augustus/) was run on the repeat-masked assembly. Finally, the results from the three approaches were integrated to generate the gene predictive models by Augustus v3.2.3. We annotated the rRNA, tRNA, and noncoding RNAs with barrnap (https://github.com/tseemann/barrnap), tRNAScan-SE v2.0 [58] and RfamScan v14.2 (http://eggnogdb.embl.de), respectively.

Genes functions were predicted by two methods. First, we used blat v36 [59] to obtain homology and similarity searches in the orthologous gene database, using a E-value cutoff of 1e-05 and an identity value of 30%. Second, domain similarity prediction strategy was performed for exploring the conserved amino acid sequences, motifs and domains with InterProScan v5.14–53.0 (http://www.ebi.ac.uk/InterProScan). (Additional details on gene function prediction are available in Note S3, see online supplementary material).

### Phylogenomic analyses for Ericales

To understand the evolutionary history within the Ericales clade, we selected 14 species (Table S19, see online supplementary material) to construct the orthogroups using OrthoFinder v2.3.1 [60]. The longest transcripts for each coding gene were chosen. MUSCLE v3.8.3.1 [61] was used for aligning the amino acid sequences from 1282 low-copy orthologs, which are shared by a minimum of 90% of species having single-copy orthologs (Table S20**,** see online supplementary material). We concatenated the alignment and constructed the maximum likelihood phylogenic species tree by IQ-TREE v1.6.7 [62] with the bootstrapped 1000 times and best model JTT + F + R5 (Fig. S17, see online supplementary material). For a coalescent-based approach, we constructed the gene trees for individual low-copy orthogroups by 1000 bootstrapping replicates, and each node of gene trees was checked with the bootstrap support (BS) cutoff value of 50% and those gene trees were used to perform coalescent-based phylogenetic inference by ASTRAL-Pro v1.1.2 [63] with local posterior probability (LPP). Finally, both concatenation and coalescent-based approaches inferred the consistent topology with high BS values and LPP values. The divergence times were inferred by MCMCTree module in PAML package [64] using *Nyssa sinensis* [65] as the outgroup based on codon alignment of 319 single-copy genes. Fossils of crown node of *Ericales* (89 Mya) [66] and crown node of *Rhododendron* (56 Mya) [66] were used to calibrate the age of the phylogenic tree, and timing of the tree root (105 Ma) was calibrated from the TimeTree online database (http://www.timetree.org/). Details are available in Note S4 (see online supplementary material).

### Pseudogene and genome duplication

Pseudogenes are genomic sequences that bear a similarity to protein coding genes and are unable to produce functional proteins, which are valuable resources to learn about gene and genome evolution. We used a homology searching algorithm to distinguish pseudogene sequences and annotate their chromosomal locations, sequences, and the parental coding genes by package PseudoPipe [67] with default settings. High-quality pseudogenes were identified by the following cutoffs: (i) amino acid sequence identity >30% between the pseudogene and parent gene; and (ii) the parent gene covers 50% of the pseudogene.

We investigated the genome-wide gene duplications pattern by examining both pseudogenes and protein coding genes. Briefly, the duplicated genes were annotated from five types of gene duplication models using DupGen_finder [68], including: whole-genome duplication (WGD), tandem duplication (TD), proximal duplication (less than 10 gene distance on the same chromosome: PD), transposed duplications (TRD), or dispersed duplications (DSD). Subsequently, we calculated the *K*s (numbers of substitutions per synonymous site), *K*a (numbers of substitution per nonsynonymous site), and *K*a/*K*s values for duplicate pairs with KaKs_Calculator v2 [69].

### Whole genome comparison

Here, we performed the whole genome assembly pair-wise alignment using SyRI v1.3 79 [70] with *R. simsii* genome as reference. The structural variations (inversion, translocation, and duplication), and the local sequence differences (SNP, indel, and so on) were determined with the pair-wise comparison for *R. simsii* and *R. molle*. We also performed the homolog-guided self- and intergenomic alignment of two assemblies using MCSCANX. The *K*s value was calculated for identified paralogous and orthologous gene pairs.

### Metabolomic assessment

All corolla samples mentioned above were used for performing the liquid chromatography-mass spectrometry (LC–MS) and data analyses, with 50 mg of each tissue. Mass spectra were obtained by liquid chromatography system coupled to an Impact II UHR-QqTOFSystem with heated electrospray ionization source. For data acquisition, we used the progenesis QI Data Processing Software for noise subtractions, retention time alignments, peak pickings, and identifying the possible metabolites. The detected metabolites were annotated against a combined database comprising the theoretical adducts from Human Metabolome Database (HMDB), LipidMaps, and METLIN. For quality control, the ion peaks with a missing rate of more than 50% within the group were eliminated, and the 0 values were replaced with half of the minimum value. Additionally, metabolites with a score of less than 30 out of 60 were removed. We combined the positive and negative data for principle component analysis (PCA) and (orthogonal) partial least-squares-discriminant analysis (O)PLS-DA by R ropls package [71], respectively. Differential metabolites with variable importance in the projection (VIP) values greater than one were taken into consideration.

### Transcriptomic analyses

We isolated the total RNAs from 25 corolla tissues mentioned above and we eluted the RNA in 50 μL RNase-free water per reaction and determined the quality on an Agilent 2100 BioAnalyzer (Agilent Tech, Santa Clara, CA, USA). All of the 150 bp PE messenger RNA sequencing libraries were prepared and then sequenced on the Illumina HiSeq X Ten platform. We removed the low-quality bases (30 < score) and adapter sequence from raw Illumina reads. Clean reads were mapped against the final genome assembly by HiSat2 v2 with default settings. Unique mapped reads were maintained to calculate the counts and transcripts per kilobase million (TPM) table by featureCounts [72]. We identified the significant differentially expressed genes (DEGs) between any two points among the five flowering time points using DEseq2 [73], with a log2 fold-change (FC) cutoff value of 1 and 0.05 > FDR. More details on DGEs are shown in Fig. S18, see online supplementary material.

### Flower color genes and transcription factors

Flower coloration is determined by the accumulation and metabolism of anthocyanins, carotenoids, chlorophylls, and flavonols. The enzymic genes were annotated from the biosynthetic pathways associated with the carotenoids/chlorophylls/flavonols/anthocyanins by querying the Plant Metabolic Network [74] with the Ensemble Enzyme Prediction Pipeline (E2P2) package v3.1 (https://gitlab.com/rhee-lab/E2P2). Targeted pathways were constructed with PlantCyc (https://plantcyc.org/) and validated with Semi Automated Validation Infrastructure v3.02 [74]. PlantRegMap [75] was used for identifying the transcription factors in *R. molle* (Table S21, see online supplementary material). To clarify the evolutionary relationships between CHS proteins from *R. simsii* and *R. molle*, we aligned the sequences of 25 CHS proteins using AtCHS (AT5G13930) as an outgroup. The conserved motifs of CHS proteins identified by MEME v5.4.1 (https://meme-suite.org/meme), and conserved domains and important sites were predicted by InterProScan v87.0 (http://www.ebi.ac.uk/interpro). Additional details were shown in Note S5, see online supplementary material. We compared the size of the same enzymatic gene families between *R. simsii* and *R. molle* after removing 33 unexpressed enzymatic genes (TPM = 0) across five flowering stages (Table S22, see online supplementary material).

### Time*-*ordered gene co*-*expression networks (TO-GCNs)

To investigate the regulatory mechanism of flower pigmentation, we constructed TO-GCNs [76] for yellow flower (*R. molle*-specific TO-GCN) and red flower (*R. simsii*-specific TO-GCN) and a consensus TO-GCN between the two networks. Gene annotation and expression profiles of *R. simsii* were explored under the database http://rhododendron.plantgenie.org/. A total of 34 953 potential orthologs (the reciprocal and single-side best hits) between the two genomes were inferred with GeneTribe [77] and a pair of orthologous genes were merged as single node in consensus TO-GCN. We defined a gene as expressed DEG if it is significantly differentially expressed between any two points among the five flowering time points (T1–T5) and its average TPM is greater than 0.5. The expressed DEGs were used for subsequent TO-GCN construction. Pearson’s product moment correlation coefficient cutoff was employed of 0.81 for each pair of TFs and all coding genes. As the initial nodes, two bHLH transcription factors (*Rhmol04G0082400* and *Rhsim13G0024200*) which were highly expressed at the first time point and monotonically decreased until T5 were selected to generate TO-GCNs by MFSelector [78]. Three TO-GCNs under the ‘C1 + C2+’ mode were combined for a large network and then visualized using Cytoscape [79]. The degrees of the nodes (genes) were calculated as the number of connections or edges the nodes have to other nodes.

Hub genes were delimited as nodes with a high degree in networks. A total of 2000 bp upstream sequences of the genes were extracted to predict TF binding sites under the condition of *P*-value <1e-^4^ by FIMO [80]. More details on TO-GCNs construction are available in Fig. S18, see online supplementary material.

## Acknowledgements

This work was supported by grants from the Strategic Priority Research Program, Chinese Academy of Sciences (Grant No. XDA23080000) and Second Tibetan Plateau Scientific Expedition and Research (STEP) program (2019QZKK0502).

## Author contributions

F.-S.Y and J.-F.M planned and designed the research. S.N., S.-W.Z, T.-L.S., W.Z., R.-G.Z., X.-C.T., J.-F.G., X.-M.Y., Y.-T.B., Z.-C.L, L.K., H.-Y.M., Z.-Y.C., and H.L. performed experiments, conducted fieldwork, analysed data etc. J.-F.M., F.-S.Y. and S.N. wrote the manuscript; I.P. and Y.A.E.-K. were involved in finalizing the manuscript draft.

## Data availability

The genome assembly, annotation, and raw sequence data have been deposited in NCBI (https://www.ncbi.nlm.nih.gov/) with the accession number JAKSEO000000000 (BioProject: PRJNA804375). The whole genome assembly, gene and repeat element annotations have been deposited in the Genome Warehouse in the National Genomics Data Center (https://ngdc.cncb.ac.cn/gwh/) under BioProject number PRJCA012078.

## Conflict of interests

The authors declare no competing interests.

## Supplementary data

Supplementary data is available at *Horticulture Research* online.

## Supplementary Material

Web_Material_uhac241
