# Supplementary material for: Gapless genome assembly of azalea and multi-omics investigation into divergence between two species with distinct flower color
Source: Hortic Res. 2022 Oct 26;10(1):uhac241. doi: 10.1093/hr/uhac241 (PMC9832866; doi:10.1093/hr/uhac241)
Supplement: Web_Material_uhac241 [file web_material_uhac241.zip › 03_Revised_SupplementaryFiles.pdf]

Note S1. Genome sequencing

Oxford Nanopore Technologies (ONT), Illumina and Hi-C sequencing

To gain an accurate assembly with high continuity, techniques from different sequencing platforms were employed, including ONT, Illumina, and Hi-C sequencing. We collected the fresh young leaves from one individual plant for three different types of sequencing.

For ONT sequencing, total DNA was extracted using the cetyltrimethylammonium bromide (CTAB) method with a Genomic DNA kit (Qiagen, Hilden, Germany). After purification and quantification, adaptors were ligated using the LSK109 kit. Then, sequencing libraries with ~20 kb DNA inserts were prepared and sequenced on a GridION X5 instrument with 1 cell.

For Illumina sequencing, we extracted total DNA using the DNeasy Plant Mini Kit (QIAGEN, Inc.), and then DNA was purified using the Mobio PowerClean Pro DNA Clean-Up Kit (MO BIO Laboratories, Inc.). 150 bp paired-end (PE) libraries were prepared using the DNAseq Library Index Kit and sequenced on an Illumina HiSeq X Ten platform. Short reads were processed with fastp (version0.19.3)[1] to remove adapter sequences, leading and trailing bases with a quality score below 20, and reads with an average per-base-quality of 20 over a 4-bp sliding window.

For Hi-C sequencing, DNA was extracted and crosslinked, and libraries were constructed following a previously published study[2]. The Hi-C libraries were then amplified and sequenced on the Illumina HiSeq X Ten platform with 150 bp paired-end (PE) reads.

A total of 2.4 million ONT long reads were generated, yielding 52.07 Gb (roughly 80× coverage of the assembled genome) with an average read length of 21,761 bp, N50 of 27,299 bp and max read length of 180,375 bp (**Table S2**). After quality control, we obtained 321.83 million clean Illumina short-read reads, (~48.22 Gb and roughly 80× coverage of the genome) (**Table S2**). For the Hi-C sequencing, we obtained 193.78 Gb of 1291.87 million reads, about 200× coverage of the assembled genome (**Table S2**).

RNA sequencing

RNA sequencing was applied on five samples of the five types of tissue (that is young leaves, stamens, young stems, pistils and petal lower lips) for gene annotation, further 25 corolla samples at five developmental time points were collected and prepared for

RNA sequencing, with five biological replicates per point, for time-ordered gene expression study of flower coloration. All of the frozen tissues were ground with a mortar and a pestle. Messenger RNA was isolated using the NEBNext Poly(A) mRNA Magnetic Isolation Module. In total, we constructed 30 libraries using the NEBNext Ultra RNA Library Prep Kit and sequenced on an Illumina HiSeq X Ten machine in 150 bp PE mode.

Finally, we obtained a total of 239.59 million raw reads (~ 35.94 Gb, from the five tissue types) for gene annotation (**Table S7**) and 1663.023 million raw reads (about 249.452 G, from a total of 25 corolla samples corresponding to the five flower developmental time points) (**Table S17**).

Note S2. Genome survey and assembly

Genome survey

A total of 49.12 Gb of PCR-free Illumina paired-end sequencing data were used for the genome survey with a *K*-mer-based approach. The k-mer distribution was then investigated using Jellyfish v2.2.10 [3] and genome size was approximately estimated at 640 Mb. We also used GenomeScope [4] to estimate the overall genome characteristics, including heterozygosity rate, and unique content rate. And we found the sequenced individual has low heterozygosity (~0.78%), and unique content rate (34.5%).

Assembly of chloroplast and mitochondrial genomes

The clean Illumina reads were used for draft assemblies of chloroplast and mitochondrial genomes with GetOrganelle[5] And preceding the filtered and corrected ONT reads were mapped on both organelle draft assemblies by minimap2 (version 2.11-r797)[6]. All mapped reads were extracted for the following assemblies. Firstly, we used Canu (version 1.7) and SMARTdenovo (version 1.0.0) (https://github.com/ruanjue/smartdenovo) to generate two primary assemblies.

Finally, the assembled mitochondrial genome gave three linear scaffolds of 1,103,744 bp, with 45.70% average GC content. And we also assembled the complete chloroplast genome with length of 196,650 bp and an GC content of 36.05% (**Table S3**).

De novo nuclear genome assembly

The *de novo* genome assembly employed the following three steps: primary assembly with Overlap-Layout-Consensus approach, Hi-C scaffolding, and polishing. Firstly, the primary assembly v0.1 was corrected and generated by NextDenovo and SMARTdenovo (-k 20) and assembly v0.2 by NextDenovo and WTDBG (version 2.1)[7] from raw ONT long reads. Then, we used the corrected reads to prepare assembly v0.3 by NextDenovo. After comparison among different primary assemblies on continuity and completeness, assembly v0.3 (reasonably sized assembly, fewest contigs) was chosen as optimal for further polished with high quality Illumina reads with one round of pilon (version 1.22) (http://github.com/broadinstitute/pilon) to produce assembly v1.0.

Subsequently, valid Hi-C data were processed together with assembly v1.0 by 3D-DNA pipeline (version 180922) (https://github.com/theaidenlab/3d-dna) to produce primary scaffolds. These scaffolds were roughly spilt by Juicebox (version 1.8) (https://github.com/aidenlab/Juicebox), and each scaffold was processed by 3D-DNA (version 180922). Afterwards, we elaborately optimized the new scaffolds by removing error insert, bound, order, and mis-join. After scaffold adjustment, we merged chromosome-level scaffolds, scattered contigs, and organelle genomes for further gap closing and polishing. Gaps were closed by LR_Gapcloser (version 1.1) (https://github.com/CAFS-bioinformatics/LR_Gapcloser) with corrected ONT long reads and three rounds of pilon polishing with filtered Illumina short reads.

For the polished assembly, we recognized and selectively removed debased contigs, such as redundant contigs (i.e. same region in homologous chromosomes) with Redundans (version 0.13c)[8], and contigs representing either low coverage below 10-fold or high non-coverage above 60%. Thereafter, the filtered assembly was aligned to the NT database with blastn (version 2.2.28+)[9] (coverage of 90%), and we confirmed that the final assembly v1.1 was not polluted by cross-contamination (**Table S18**). For 13 pseudochromosomes, telomeres were identified by regions where the characteristic repeat motif (TTTAGGG) was repeated more than 5 times. The telomeres were identified at the ends of six pseudochromosomes (chr03, chr04, chr06, chr07, chr08 and chr09) and at one end of five pseudochromosomes (chr01, chr02, chr10, chr12, and chr13), respectively. And telomeres were not identified on pseudochromosomes chr05 and chr11 (**Fig. S4**).

Quality evaluation

In the final assembly, we obtained a chromosome-level genome size of 653.46 Mb, which have better contiguity than any published Rhododendron genomes, with only nine gaps, 34 contigs (contig N50 = 45 Mb; longest contig = 64 Mb) and 25 scaffolds (scaffold N50 = 53 Mb and longest scaffold = 68 Mb). And 13 pseudo-chromosomes were assembled that covered ~99.48% of the assembled genome (**Table S1**). And seven pseudochromosomes (chr02, chr05, chr07, chr08, chr09, chr12 and chr13) were represented with gap-free and two (chr07 and chr08) of them were entirely reconstructed in a single scaffold from telomere to telomere (**Fig. 1a** and **Fig. S4**). Only nine gaps were localized to six pseudochromosomes (chr01, chr03, chr04, chr06, chr10 and chr11), including the four gaps within extremely repetitive pericentromeric regions in one pseudochromosome (chr11) (**Fig. 1a**).

We assessed the completeness and continuity of our assemblies in several ways: (i) a high LTR Assembly Index (LAI) score of 14.9[10] indicated that the assembly has reached the reference level; (ii) 93.4% gene completeness were found by mapping 1,440 Benchmarking Universal Single Copy Orthologs (BUSCO)[11] genes to the assembly (**Table S3**); (iii) a high mapping rate of properly paired reads of 97.3% were obtained when all clean Illumina reads mapped to final assembly with BWA-MEM (https://github.com/lh3/bwa); (iv) a high overall reads mapping rate of 99.5% was observed for mapping ONT long reads by minimap2 (version 2.11-r797). (v) Using RNA-seq data from five tissues, a total 96.0 % of the sequences could be mapped onto the genome assembly by HiSat2 (version 2.1.0) (https://github.com/infphilo/hisat2) (**Table S3**).

For base accuracy assessment of our assembly, after Illumina reads from genome sequencing mapped to the final assembly, we gained a low heterozygosity of ~0.52% and a very low single base error rate (~0.0036%) based on SNPs calling with SAMtools[12]. The good chromatin interactions were shown by Hi-C reads mapped onto the final assembly by Juicer (https://github.com/aidenlab/juicer) (**Fig. S1**).

Note S3. Genome annotation

Transposable element and other repeat annotation

Repeat sequences were identified and classified through a combination of *de novo* and homology-based approaches. *de novo* prediction was carried out using RepeatModeler

(version 1.0.8) (http://www.repeatmasker.org). Outputs generated by RepeatModeler were collected as a repeat library. Intact LTRs were *de novo* identified using LTR_retriever[13] and used to construct another repeat library. The two libraries were merged for homology-based repeats characterization by RepeatMasker v4.0.7 (rmblast-2.2.28) (http://www.repeatmasker.org). A total of 668,841 repeat elements (34,9487,787 bp) were predicted occupying 53.48% of the entire genome. Most of the repeat sequences are long terminal repeat (LTR)-type retrotransposons (RTs) (277,144,592 bp), which account for 42.42%; 2.70% is DNA transposons (17,668,757 bp), and the long interspersed nuclear elements (LINE) account for only 1.37%. Among LTR-RTs, the families *Gypsy* and *Copia* accounted for 27.86 and 4.9 % of the genome, respectively.

We further examined the proliferation, age dynamics, gene proximity, birth and death of LTR-RTs. LTRharvest[14] and LTRdigest[15] were used for *de novo* prediction of candidate LTR-RT by criterion: 1) 1-15 kb from other candidates; 2) flanked a pair of putative LTRs; 3) range from 100 to 3,000 bp; 4) similarity >80%. Further, the identified LTR-RTs were classified according to the internal organization of the coding domains using REXdb (Viridiplantae v3.0) [16] with LAST v963 (http://last.cbrc.jp) alignment tool[17]. If a LTR-RT candidate possessed a complete Gag-Pol protein sequence, it was retained as an intact LTR-RT (*I*). We identified LTR paralogs with sequence similarity to the intact LTR-TRs (E-value < 1e−10, overlap length >90%, identity >90%). The LTR paralogs that showed at least 50% Gag-Pol coverage by one side of the flanking sequence and 30% identity (E-value < 1e−8) were considered to be truncated LTR-RTs (*T*). And LTR-RTs lacking Gag-Pol up- and downstream of the LTRs were retained as solo-LTRs (*S*). Superfamily classifications within the *Gypsy* and *Copia* classes are provided in **Table S12**.

We estimated insertion time (T) of a intact LTR-RT based on the divergence between the 5' LTR and 3'-LTR of the same transposon using mafft (version 7.221)[18] following equation T = K/2r, with mutation rate r = 1.5×10^{-8} per site per year[19]. Here the Kimura two-parameter method[20] was employed to calculate the divergence of two LTRs from one intact LTR retrotransposons (*K*) (**Fig. S9**). Meanwhile, relationships of the gene proximity and the insertion time were examined by calculating the distances between intact LTR-RTs to a closest gene.

To understand the relationships among individual LTR-RTs, 5' LTR sequences of all LTR-RTs were compared against each other with blastn using Silix (version

1.2.9)[21]. Two LTRs were assigned to the same cluster if they mutually covered at least 70% of their lengths with an identity of at least 60% between them. Solo-LTRs (*S*) and truncated LTR-RTs (*T*) were also mapped to the same cluster containing 5' LTRs from the most similar intact LTR-RTs (*I*). Furthermore, ratios of solo-LTR-RTs and truncated LTR-RTs, respectively, to intact LTR-RTs (*S:I*, *T:I*) as well as their sums were assessed to study the removal rates of LTR-RTs over the past several million years. Then, we evaluated LTR-RT deletions using proportions of clusters with *S:I* values greater than 3. For an interspecific comparison, we also conducted the same LTR-RTs analyses for *R. simsii* genome (**Table S13**).

Gene structural and functional annotation

A total of 35.94 G raw RNA-seq reads were processed using Trimmomatic (version 0.36)[22] and Cutadapt (version 1.13)[23]. And ~34.91 G clean reads were aligned to the genome assembly with HiSat2 (version 2.1.0). Here, we constructed transcriptome assemblies following *de novo* mode with Trinity (version 2.0.6)[24], and reference genome-guided mode with StringTie (version 1.3.5)[25] and Trinity (version 2.0.6). All assemblies were combined and then refined them by CD-HIT (version 4.6)[26]. Finally, 124,785 unique transcripts were predicted as expressed sequence tag (EST) evidence for gene prediction.

Coding gene models were predicted by MAKER2 pipeline (version 2.31.9)[27]. The repeat-masked genome was used in both, evidence-based and *ab initio* gene prediction, strategies. For evidence-based gene prediction, protein homology evidence was generated by CD-HIT (95% identity and 95% coverage) with clustered the protein sequences from the genome of *Arabidopsis thaliana* [28], *Actinidia chinensis* [29], *Rhododendron simsii* [30], *Rhododendron williamsianum* [31], *Vaccinium corymbosum* [32], and *Rhododendron delavayi* [33]. Subsequently, the EST and protein evidences were aligned to the repeat-masked genome and then polished by Exonerate (version 2.4.0)[34]. The *ab initio* gene prediction was compared to evidence-based gene models to revise the gene predictions with AUGUSTUS (version 3.3)[35, 36]. Finally, a reliable annotated gene set were generated after removing genes which had abnormity open reading frames (ORFs) or were too short (<= 50 aa).

Among non-coding RNAs (ncRNAs), tRNAs, ribosomal RNAs (rRNAs) and other ncRNAs were annotated using tRNAscan-SE (version 1.3.1)[37], RNAMMER (version

1.2)[38], and RfamScan (version 9.1) (http://eggnogdb.embl.de/), respectively.

Predicted gene models were aligned to proteins in eight protein databases: (i) NR (https://www.ncbi.nlm.nih.gov/), (ii) Swiss-Prot protein database[39], (iii) Translated EMBL-Bank (as part of the International Nucleotide Sequence Database Collaboration TrEMBL[39] , (iv) Pfam[40], (v) Cluster of Orthologous Groups for eukaryotic complete genomes (KOG) database, (vi) KEGG (the Kyoto Encyclopedia of Genes and Genomes, Orthology) database[41], (vii) GO[42], and (8) UniProt database[39] with blat (version 36)[43] (identity > 30% and E < $1e^{-05}$). For domain similarity predictions, the predicted protein sequences were annotated using InterProScan (version 5.27-66.0) (http://www.ebi.ac.uk/InterProScan) with default parameters. we concatenated the annotations derived all strategies to obtain the final gene functional annotations.

We finally predicted 40,023 protein-coding genes, 477 tRNAs, 98 rRNAs and an additional 1,002 ncRNAs (**Table S1** and **S8**). And most of predicted genes (91.41%) could be annotated with the outcomes for at least one of the protein-related databases (**Table S10**).

Note S4. Gene families

In order to ascertain the evolutionary history of Ericales, we selected 14 species to construct gene families or orthogroups, including 13 Ericales (*Actinidia chinensis, Aegiceras corniculatum, Roridula gorgonias, Rhododendron delavayi, Rhododendron ovatum, Rhododendron williamsianum, Rhododendron griersonianum, Rhododendron simsii, Clethra arborea, Camellia sinensis, Vaccinium macrocarpon* and *Diospyros oleifera*), and one Cornales *Nyssa sinensis* (**Table S22**). The longest transcript isoform for each locus was selected for putative gene families identification using OrthoFinder (version 2.3.1)[44]. In total, 431,542 genes (94.0% of the total sequences) from the selected 14 species were clustered into 29,747 gene families. And 7,478 families were shared among all these genomes, and 5,069 families were species-specific. For *R. molle*, a total of 37,572 genes (93.9% of all 40,023 coding genes) were clustered into 18,767 families, and 395 families (1,361 genes) were found to be *R. molle* -specific (**Table S20**).

1,282 low-copy families with minimum of 92.9% of species having single-copy genes in any orthogroup were determined for constructing a phylogenetic tree with *Nyssa sinensis* as outgroup. Amino acid sequence matrices were created by MUSCLE

(version 3.8.31)[45] using default settings, and concatenated amino acid sequences were trimmed using trimAI (version 1.2) (trimal -gt 0.8 -st 0.001 -cons 60)[46]. And the maximum likelihood (ML) tree was constructed using IQ-TREE (version 1.6.7)[47] with the optimal sequence evolution model (-m JTT+F+R5), Shimodaira-Hasegawa-like approximate likelihood-ratio PAL2NAL test (SH-aLRT, -alrt 1000)[48], and ultrafast bootstrapping (-bb 1000)[49, 50]. Every nodes of the ML tree were well-supported with the bootstrapping value of 100.

We also used coalescent-based approach to infer the phylogenetic relationships within Ericales with 1,282 low-copy orthogroups. The individual gene trees were constructed by 100 rapid bootstrapping replicates using IQ-TREE (version 1.6.7) [47] with the optimal sequence evolution model (-m MF). we checked the bootstrap support (BS) values for the nodes. The individual gene trees with BS > 50% were then used by ASTRAL-Pro (version 1.1.2) [51] with local posterior probability (LPP). And a same topology was inferred by both concatenate and coalescent-based approaches, and used as reference tree for the further analyses.

The divergence time were estimated with two files: the ML tree and trimmed amino acid alignments of 319 single-copy families using MCMCTREE implemented in PAML v4.9h[52] with the following settings: the approximate likelihood method with the independent substitution rate, JC69 substitution model, 2.1e6 iterations and 1e5 iterations discarded as burn-in. The phylogeny was calibrated using two fossil dating points and a soft bound at three nodes: (i) the crown node of *Rhododendron* (56 Mya)[53], (ii) the crown node of ericales (89.8 Mya)[54] and (iii) ericales-cornales (105–119 Mya) with TimeTree (http://timetree.temple.edu/). We found *R. molle* diverged from the most recent common ancestor of *R. delavayi, R. griersonianum* and *R. williamsianum* around 45.29 Mya, following the divergence of *R. simsii* and *R. ovatum* around 52.21 Mya (**Fig. S17**).

Among all transcription factors (TFs), MYBs[55, 56], basic helix-loop-helix (bHLH) proteins[57, 58], WD40s[59], and their associated transcriptional complexes have been shown to regulate multiple enzymatic steps crucial in the production of flavonoids. To identify the potential members, 531 *Arabidopsis* protein sequences for each of these three TF families (MYB: 132; bHLH: 169; WD40: 230) were used as reference with blastp. In addition, we used PlantRegMap[60-62] to identify TFs as supporting evidence of prediction. Furthermore, all obtained protein sequences were manually inspected with SMART (http://smart.embl-heidelberg.de/) and the domains

presences were confirmed in NCBI Batch Web CD-Search Tool (https://www.ncbi.nlm.nih.gov/Structure/bwrpsb/bwrpsb.cgi). Finally, we identified 2,009 TFs with 59 TF families, yielding 118 *MYB*, 164 *bHLH* and 253 *WD40* genes in *R. molle*, respectively (**Table S21**).

Note S5. Flower pigmentation genes

Here, we constructed the metabolic pathways that could be linked to the formation of yellow flowers of *R. molle*: chlorophyll, carotenoid, anthocyanin and flavonol (**Fig. 2**, **3a** and **Fig. S12**). In *R. molle*, we identified 52, 64, 90 and 104 genes (**Table S15**) encoding enzymes functioning in the carotenoid, chlorophyll, flavonol and anthocyanin biosynthesis, respectively. The formation of yellow flower or fruit is generally accompanied by biosynthesis of carotenoids and flavonol, coupled with degradation of chlorophyll [63]. In *R. molle*, two single-copy genes (Rhmol07G0115200; Rhmol10G0271900) were discovered encoding STAY-GREEN (SGR) protein and phytoene synthase (PSY), as major rate-controlling enzymes for chlorophyll degradation and carotenoid biosynthesis (**Fig. 2)**. Furthermore, we predicted seven genes encoding flavonol synthase (FLS) and multiple-copy genes (18) for flavonoid 3′-hydroxylase/flavonoid 3′,5′-hydroxylase (F3′H/ F3′5′H) playing major roles in biosynthesis of flavonol (**Fig. 3a** and **Fig. S12**).

Meanwhile, we collected the identified pathways on carotenoid and anthocyanin/flavonols of *R. simsii* [30], and built the chlorophyll metabolic pathway (**Fig. S13**). In the genome of *R. simsii*, there were a total of 54, 58, 112 and 125 genes (**Table S15**) encoding enzymes functioning in the chlorophyll, carotenoid, flavonol and anthocyanin biosynthesis, respectively.

Supplementary References

1. Chen, S., et al., *fastp: an ultra-fast all-in-one FASTQ preprocessor.* Bioinformatics, 2018. **34**(17): p. i884-i890.
2. Duan, Z., et al., *A three-dimensional model of the yeast genome.* Nature, 2010. **465**(7296): p. 363-367.
3. Marçais, G. and C. Kingsford, *A fast, lock-free approach for efficient parallel counting of occurrences of k-mers.* Bioinformatics, 2011. **27**(6): p. 764-770.
4. Vurture, G.W., et al., *GenomeScope: fast reference-free genome profiling from short reads.* Bioinformatics, 2017. **33**(14): p. 2202-2204.
5. Jin, J.-J., et al., *GetOrganelle: a fast and versatile toolkit for accurate de novo assembly of organelle genomes.* Genome Biology, 2020. **21**(1): p. 241.
6. Li, H., *Minimap2: pairwise alignment for nucleotide sequences.*

Bioinformatics, 2018. **34**(18): p. 3094-3100.
7. Ruan, J. and H. Li, *Fast and accurate long-read assembly with wtdbg2.* Nature Methods, 2020. **17**(2): p. 155-158.
8. Pryszcz, L.P. and T. Gabaldón, *Redundans: an assembly pipeline for highly heterozygous genomes.* Nucleic Acids Research, 2016. **44**(12): p. e113.
9. Boratyn, G.M., et al., *Domain enhanced lookup time accelerated BLAST.* Biol Direct, 2012. **7**: p. 12.
10. Ou, S., J. Chen, and N. Jiang, *Assessing genome assembly quality using the LTR Assembly Index (LAI).* Nucleic Acids Research, 2018. **46**(21): p. e126.
11. Simao, F.A., et al., *BUSCO: assessing genome assembly and annotation completeness with single-copy orthologs.* Bioinformatics, 2015. **31**(19): p. 3210-3212.
12. Li, H. and R. Durbin, *Fast and accurate short read alignment with Burrows-Wheeler transform.* Bioinformatics, 2009. **25**(14): p. 1754-60.
13. Ou, S. and N. Jiang, *LTR_retriever: a highly accurate and sensitive program for identification of long terminal repeat retrotransposons* Plant Physiology, 2018. **176**(2): p. 1410-1422.
14. Ellinghaus, D., S. Kurtz, and U. Willhoeft, *LTRharvest, an efficient and flexible software for de novo detection of LTR retrotransposons.* BMC Bioinformatics, 2008. **9**: p. 18.
15. Steinbiss, S., et al., *Fine-grained annotation and classification of de novo predicted LTR retrotransposons.* Nucleic Acids Res, 2009. **37**(21): p. 7002-13.
16. Neumann, P., et al., *Systematic survey of plant LTR-retrotransposons elucidates phylogenetic relationships of their polyprotein domains and provides a reference for element classification.* Mob DNA, 2019. **10**: p. 1.
17. Kielbasa, S.M., et al., *Adaptive seeds tame genomic sequence comparison.* Genome Res, 2011. **21**(3): p. 487-493.
18. Katoh, K. and D.M. Standley, *MAFFT multiple sequence alignment software version 7: improvements in performance and usability.* Molecular Biology and Evolution, 2013. **30**(4): p. 772-780.
19. Koch, M.A., B. Haubold, and T. Mitchell-Olds, *Comparative evolutionary analysis of chalcone synthase and alcohol dehydrogenase loci in Arabidopsis, Arabis, and related genera (Brassicaceae).* Molecular Biology and Evolution, 2000. **17**(10): p. 1483-1498.
20. Kimura, M., *A simple method for estimating evolutionary rates of base substitutions through comparative studies of nucleotide sequences.* J Mol Evol, 1980. **16**(2): p. 111-20.
21. Miele, V., S. Penel, and L. Duret, *Ultra-fast sequence clustering from similarity networks with SiLiX.* BMC Bioinformatics, 2011. **12**: p. 116.
22. Bolger, A.M., M. Lohse, and B. Usadel, *Trimmomatic: a flexible trimmer for Illumina sequence data.* Bioinformatics, 2014. **30**(15): p. 2114-2120.
23. Martin, M., *Cutadapt removes adapter sequences from high-throughput sequencing reads.* EMBnet.journal, 2011. **17**(1).
24. Grabherr, M.G., et al., *Full-length transcriptome assembly from RNA-Seq data without a reference genome.* Nat Biotechnol, 2011. **29**(7): p. 644-652.
25. Pertea, M., et al., *StringTie enables improved reconstruction of a transcriptome from RNA-seq reads.* Nature Biotechnology, 2015. **33**(3): p. 290-295.
26. Fu, L., et al., *CD-HIT: accelerated for clustering the next-generation sequencing data.* Bioinformatics, 2012. **28**(23): p. 3150-2.

27. Holt, C. and M. Yandell, *MAKER2: an annotation pipeline and genome-database management tool for second-generation genome projects.* BMC Bioinformatics, 2011. **12**: p. 491.
28. Lamesch, P., et al., *The Arabidopsis Information Resource (TAIR): improved gene annotation and new tools.* Nucleic Acids Research, 2012. **40**(Database issue): p. D1202-D1210.
29. Wang, J.P., et al., *Two Likely Auto-Tetraploidization Events Shaped Kiwifruit Genome and Contributed to Establishment of the Actinidiaceae Family.* iScience, 2018. **7**: p. 230-240.
30. Yang, F.S., et al., *Chromosome-level genome assembly of a parent species of widely cultivated azaleas.* Nature Communications, 2020. **11**(1): p. 5269.
31. Soza, V.L., et al., *The Rhododendron genome and chromosomal organization provide insight into shared whole-genome duplications across the heath family (Ericaceae).* Genome Biology and Evolution, 2019. **11**(12): p. 3353-3371.
32. Colle, M., et al., *Haplotype-phased genome and evolution of phytonutrient pathways of tetraploid blueberry.* Gigascience, 2019. **8**(3): p. giz012.
33. Yan, H., et al., *The draft genome assembly of Rhododendron delavayi Franch. var. delavayi.* GigaScience, 2017. **6**(10): p. 1-11.
34. Slater, G.S. and E. Birney, *Automated generation of heuristics for biological sequence comparison.* BMC Bioinformatics, 2005. **6**: p. 31.
35. Keller, O., et al., *A novel hybrid gene prediction method employing protein multiple sequence alignments.* Bioinformatics, 2011. **27**(6): p. 757-763.
36. Stanke, M., et al., *Using native and syntenically mapped cDNA alignments to improve de novo gene finding.* Bioinformatics, 2008. **24**(5): p. 637-44.
37. Lowe, T.M. and S.R. Eddy, *tRNAscan-SE: a program for improved detection of transfer RNA genes in genomic sequence.* Nucleic Acids Res, 1997. **25**(5): p. 955-64.
38. Lagesen, K., et al., *RNAmmer: consistent and rapid annotation of ribosomal RNA genes.* Nucleic Acids Res, 2007. **35**(9): p. 3100-8.
39. Bairoch, A. and R. Apweiler, *The SWISS-PROT protein sequence database and its supplement TrEMBL in 2000.* Nucleic Acids Res, 2000. **28**(1): p. 45-8.
40. Finn, R.D., et al., *Pfam: the protein families database.* Nucleic Acids Res, 2014. **42**(Database issue): p. D222-30.
41. Kanehisa, M. and S. Goto, *KEGG: Kyoto Encyclopedia of Genes and Genomes.* Nucleic Acids Research, 2000. **28**(1): p. 27-30.
42. Harris, M.A., et al., *The Gene Ontology (GO) database and informatics resource.* Nucleic Acids Research, 2004. **32**(Database issue): p. D258-61.
43. Kent, W.J., *BLAT--the BLAST-like alignment tool.* Genome Research, 2002. **12**(4): p. 656-664.
44. Emms, D.M. and S. Kelly, *OrthoFinder: phylogenetic orthology inference for comparative genomics.* Genome Biology, 2019. **20**(1): p. 238.
45. Edgar, R.C., *MUSCLE: multiple sequence alignment with high accuracy and high throughput.* Nucleic Acids Research, 2004. **32**(5): p. 1792-1797.
46. Capella-Gutiérrez, S., J.M. Silla-Martínez, and T. Gabaldón, *trimAl: a tool for automated alignment trimming in large-scale phylogenetic analyses.* Bioinformatics, 2009. **25**(15): p. 1972-1973.
47. Nguyen, L.T., et al., *IQ-TREE: a fast and effective stochastic algorithm for estimating maximum-likelihood phylogenies.* Molecular Biology and Evolution, 2015. **32**(1): p. 268-274.
48. Guindon, S., et al., *New algorithms and methods to estimate maximum-*

likelihood phylogenies: assessing the performance of PhyML 3.0. Systematic biology, 2010. **59**(3): p. 307-321.

49. Hoang, D.T., et al., *UFBoot2: Improving the Ultrafast Bootstrap Approximation.* Molecular Biology and Evolution, 2018. **35**(2): p. 518-522.
50. Minh, B.Q., M.A. Nguyen, and A. von Haeseler, *Ultrafast approximation for phylogenetic bootstrap.* Mol Biol Evol, 2013. **30**(5): p. 1188-95.
51. Zhang, C., et al., *ASTRAL-Pro: Quartet-based species-tree inference despite paralogy.* Molecular Biology and Evolution, 2020. **37**(11): p. 3292-3307.
52. Yang, Z., *PAML 4: phylogenetic analysis by maximum likelihood.* Molecular Biology and Evolution, 2007. **24**(8): p. 1586-1591.
53. Collinson, M.E. and P.R. Crane, *Rhododendron seeds from the Palaeocene of southern England.* Botanical Journal of the Linnean Society, 1978. **76**(3): p. 195-205.
54. Nixon, K.C. and W.L. Crepet, *Late Cretaceous fossil flowers of Ericalean affinity.* American Journal of Botany, 1993. **80**(6): p. 616-623.
55. Dubos, C., et al., *MYB transcription factors in Arabidopsis.* Trends in Plant Science, 2010. **15**(10): p. 573-581.
56. Li, X., et al., *Genome-wide identification, evolution and functional divergence of MYB transcription factors in Chinese white pear (Pyrus bretschneideri).* Plant Cell Physiology, 2016. **57**(4): p. 824-847.
57. Pires, N. and L. Dolan, *Origin and diversification of basic-helix-loop-helix proteins in plants.* Mol Biol Evol, 2010. **27**(4): p. 862-74.
58. Wei, K. and H. Chen, *Comparative functional genomics analysis of bHLH gene family in rice, maize and wheat.* BMC Plant Biol, 2018. **18**(1): p. 309.
59. Li, Q., et al., *Genome-wide analysis of the WD-repeat protein family in cucumber and Arabidopsis.* Molecular Genetics and Genomics, 2014. **289**(1): p. 103-124.
60. Tian, F., et al., *PlantRegMap: charting functional regulatory maps in plants.* Nucleic Acids Research, 2019. **48**(D1): p. D1104-D1113.
61. Jin, J., et al., *An Arabidopsis transcriptional regulatory map reveals distinct functional and evolutionary features of novel transcription factors.* Molecular Biology and Evolution, 2015. **32**(7): p. 1767-1773.
62. Jin, J., et al., *PlantTFDB 4.0: toward a central hub for transcription factors and regulatory interactions in plants.* Nucleic Acids Research, 2017. **45**(D1): p. D1040-D1045.
63. Santamour, F.S. and R.L. Pryor, *Yellow flower pigments in Rhododendron: a review for breeders.* Journal of the American Rhododendron Society, 1973. **27**(4): p. 1.

Table S1: Sequencing, assembly and annotations comparison among the nine *Rhododendron* species.

	***R. molle* v2**	***R. molle* v1**	***R. henanense***	***R. ripense***	***R. ovatum***	***R. griersonianum***	***R. simsii***	***R. delavayi***	***R. williamsianum***
**Sequencing**									
Raw Bases of WGS-long-read sequencing data (Gb)	51.97	49.48	147.11	58.80	56.67	100.56	51.15	*	*
Raw Bases of WGS-Illumina (Gb)	49.12	58.43	81.22	39.90	76.00	96.91	91.49	336.83	*
Raw Bases of Hi-C (Gb)	193.78	50.00	74.44	*	70.45	92.14	55.68	*	*
Raw Bases of mRNAseq (Gb)	35.94	*	*	*	147.80	96.91	422.15	*	*
**Assembly**									
Number of contigs	34	3,764	732	318	2,668	67	911	209,969	98,253
N50 of contigs (Mb)	44.85	0.71	2.51	2.46	1.24	33.99	2.23	0.06	0.01
N90 of contigs (Mb)	22.54	0.09	0.77	0.91	*	9.07	0.33	0.01	0.001
Number of scaffolds	25	2,536	300	78	*	48	552	193,091	10,290
N50 of scaffolds (Mb)	52.64	47.02	50.18	37.02	41.00	52.93	36.35	0.64	29.01
N90 of scaffolds (Mb)	40.41	0.10	36.47	31.13	*	37.98	30.66	0.07	0.03
Final genome size (Mb)	653.46	744.48	634.29	506.73	549.71	676.81	528.64	695.09	532.29
Estimated genome size (Mb)	640	700.18	654.12	*	528.63	750	520	697.94	*
Chromsome-scale scaffolds (Mb)	650.07 (99.48%)	621.01 (83.41%)	622.93 (98.21%)	487.37 (96.18%)	549.71 (99.05%)	663.27 (98.00%)	481.95 (91.17%)	*	*
Number of Gap	9	1228	432	241	2,654	19	359	16,878	87,963
Complete BUSCOs (%)	93.40	90.90	97.00	96.90	95.30	93.10	93.68	92.80	89.00
GC content of the genome (%)	40.41	41.14	40.85	38.98	38.84	40.78	38.91	38.32	39.06
**Annotation**									
Number of predicted genes	41,600	41,916	34,379	*	43,623	39,510	32,999	32,938	23,559

Number of predicted protein-coding genes	40,023	39,288	31,098	34,606	43,623	38,280	32,999	32,938	23,559
Complete BUSCOs (%) of protein-coding genes	93.70	*	93.80	84.60	96.10	*	93.50	87.40	79.10
Average gene length (bp)	4,492.4	4,752.73	6393.37	4036.28	4,074	5,486.7	5,089.22	4,434.22	4,628
Average CDS length (bp)	1,118.7	1,213.78	1208.23	1321.95	1,166	1,204.1	1,288.73	1,153.21	*
Average exon per transcript	5.6	*	5.25	5.12	4.8	5.8	5	4.62	5.68
Number of tRNAs	477	667	448	*	*	529	482	*	*
Number of rRNAs	98	288	2,251	*	*	173	64	*	*
Repeat sequences (Mb)	349.49 (53.48%)	472.24 (63.43%)	417.11 (65.76%)	256.00 (51.31%)	245.48 (44.71 %)	385.75 (57.00%)	250.99 (47.48%)	359.87 (51.77%)	*
LTR-RTs length (Mb)	277.14 (42.41%)	257.82 (34.63%)	321.91 (50.75%)	77.24 (15.20%)	156.03 (28.42%)	167.58 (24.76%)	89.93 (17.01%)	260.53 (37.48%)	*
Gypsy LTR-RTs length (Mb)	182.05 (27.86%)	216.28 (29.05%)	*	*	*	143.34 (21.179%)	62.90 (11.90%)	177.18 (25.49%)	*
Annotated to Swissport	18,860 (47.1%)	*	20,008 (64.34%)	*	*	20,815 (54.38%)	19,079 (57.80%)	22,693 (68.90%)	*
Annotated to PFAM	23,864 (59.6%)	*	*	*	*	23,964 (62.60%)	24,301 (73.60%)	*	*
Annotated to GO	25,853 (64.6%)	17,957 (45.71%)	15,059 (48.42%)	8,843 (25.55%)	*	14,181 (37.05%)	25,038 (75.90%)	16,471 (50.00%)	18,538 (79%)
Annotated to KO	11,102 (27.7%)	11,492 (29.25%)	9,281 (29.84%)	*	*	14,007 (36.59%)	11,506 (34.90%)	*	*

R. molle v1 represents the previously reported genome assembly of *R. molle* (Zhou et al., 2022), and *R. molle* v2 represents the genome assembly of *R. molle* in the present study. *, data not available.

Table S2: Statistics of whole genome sequencing data.

Technologies	Features	Statistics
WGS-Illumina	Raw Reads (Mb)	327.489
	Raw Bases (Gb)	49.123
	Raw Q20 (Gb)	47.964 (97.64%)
	Raw Q30 (Gb)	45.905 (93.45%)
	Clean Reads (Mb)	321.834 (98.27%)
	Clean Bases (Gb)	48.221 (98.16%)
	Clean Q20 (Gb)	47.268 (98.02%)
	Clean Q30 (Gb)	45.905 (93.92%)
	Average length (bp)	150
WGS-ONT	raw reads (Gb)	52.07
	GC content	39.49%
	A	15,583,005,644 (29.99%)
	T	15,863,534,142 (30.53%)
	G	9,833,585,438 (18.92%)
	C	10,686,691,195 (20.56%)
	Reads count	2,387,974
	Max length (bp)	180,375
	Mean length (bp)	21,761
	Min length (bp)	112
	Median length (bp)	20,000
	N10 (bp)	46,581
	N50 (bp)	27,299
	N90 (bp)	13,719
	L10	95,045
	L50	696,386
	L90	1,736,754
Hi-C	Reads (Mb)	1291.864
	Bases (Gb)	193.78
	Q20 (Gb)	187.131 (96.6%)
	Q30 (Gb)	171.996 (88.8%)
	Average length (bp)	150

Table S3: Genome quality assessment.

Features	Statistics
Size estimated with 17 *k*-mer (Mbp)	640
Mitochondrial size (bp)	1,103,744
Chloroplast size (bp)	196,650
Mitochondrial GC content	45.70%
Chloroplast GC content	36.05%
Illumina reads mapped rate	99.60%
ONT reads mapped rate	99.50%
RNA-Seq reads mapped rate	96.00%
Illumina base mapped rate	99.60%
ONT base mapped rate	99.80%
RNA-Seq base mapped rate	96.00%
Illumina 1-fold minimum genome coverage	97.90%
ONT 1-fold minimum genome coverage	99.99%
RNA-Seq 1-fold minimum genome coverage	21.60%
Illumina 5-fold minimum genome coverage	97.50%
ONT 5-fold minimum genome coverage	99.95%
RNA-Seq 5-fold minimum genome coverage	13.80%
Illumina 10-fold minimum genome coverage	96.90%
ONT 10-fold minimum genome coverage	99.90%
RNA-Seq 10-fold minimum genome coverage	10.70%
Illumina 20-fold minimum genome coverage	95.40%
ONT 20-fold minimum genome coverage	99.60%
RNA-Seq 20-fold minimum genome coverage	8.20%
SNP heterozygosity with Illumina reads	0.52%
single base error rate with Illumina reads	0.0036%
Complete BUSCOs (C)	1,345 (93.40%)
Complete and single-copy BUSCOs (S)	1,269 (88.10%)
Complete and duplicated BUSCOs (D)	76 (5.30%)
Fragmented BUSCOs (F)	21 (1.50%)
Missing BUSCOs (M)	74 (5.10%)
Total BUSCO groups searched	1,440

Table S4: Summary of centromeres on the chromosomes.

Chromosome	Start (bp)	End (bp)	Size (Mb)
chr01	21,500,001	37,500,000	16.0
chr02	25,500,001	33,000,000	7.5
chr03	19,000,001	23,000,000	4.0
chr04	28,000,001	36,500,000	8.5
chr05	18,500,001	30,500,000	12.0
chr06	18,500,001	25,000,000	6.5
chr07	23,500,001	31,000,000	7.5
chr08	19,500,001	28,000,000	8.5
chr09	16,500,001	23,500,000	7.0
chr10	23,000,001	30,500,000	7.5
chr11	1	20,000,000	20.0
chr12	13,500,001	16,500,000	3.0
chr13	20,000,001	25,500,000	5.5

Table S5: Summary of telomeres on the chromosomes.

Chromosome	Start (bp)	End (bp)	Strand	Motif number	5or3
chr01	1,089	26,990	-	419	5
chr02	52,694,979	52,734,663	+	686	3
chr03	1,262	32,767	-	951	5
chr03	40,374,031	40,410,106	+	554	3
chr04	1,715	20,296	-	476	5
chr04	67,539,628	67,572,667	+	676	3
chr06	4,193	36,422	-	597	5
chr06	53,783,221	53,827,345	+	848	3
chr07	998	24,899	-	840	5
chr07	44,826,163	44,852,354	+	861	3
chr08	161	27,112	-	608	5
chr08	48,111,505	48,136,044	+	636	3
chr09	120	26,299	-	306	5
chr09	45,043,558	45,063,763	+	356	3
chr10	1,246	26,572	-	735	5
chr12	1,137	31,678	-	787	5
chr13	1,183	33,902	-	418	5

5or3, whether the telomeres are at the 5' or 3' end of the chromosome.

Table S6: Summary of gaps on the chromosomes.

Gap ID	Chromosome	Location on chromosome	Characteristics
gap1	chr01	Distal	Close to *LTR-RT*
gap2	chr03	Distal	Close to TE
gap3	chr04	Distal	Close to *Gypsy*
gap4	chr06	Middle	Overlap with centromere
gap5	chr10	Middle	Close to centromere
gap6	chr11	Distal	Close to *Gypsy*/centromere
gap7	chr11	Distal	Close to *Gypsy*/centromere
gap8	chr11	Distal	Close to *Gypsy*/centromere
gap9	chr11	Distal	Close to *Gypsy*/centromere

Table S7: Statistics of mRNA sequencing data for gene annotation.

	Young leaves	Stamens	Young stems	Pistils	Petal lower lips	Total
Raw Reads (Mb)	41.90	43.37	40.52	45.02	68.79	239.59
Raw Bases (Gb)	6.29	6.51	6.08	6.75	10.32	35.94
Raw Q20 (Gb)	6.15 (97.91%)	6.38 (98.01%)	5.96 (98.14%)	6.62 (97.99%)	10.06 (97.50%)	*
Raw Q30 (Gb)	5.95 (94.61%)	6.18 (94.97%)	5.78 (95.13%)	6.40 (94.80%)	9.67 (93.70%)	*
Clean Reads (Mb)	40.85 (97.50%)	42.32 (97.59%)	39.49 (97.46%)	43.89 (97.48%)	66.87 (97.21%)	*
Clean Bases (Gb)	6.11 (97.28%)	6.33 (97.27%)	5.91 (97.20%)	6.56 (97.20%)	10.00 (96.95%)	*
Clean Q20 (Gb)	6.02 (98.44%)	6.24 (98.56%)	5.83 (98.67%)	6.47 (98.57%)	9.82 (98.12%)	*
Clean Q30 (Gb)	5.95 (95.26%)	6.18 (95.65%)	5.78 (95.80%)	6.40 (95.51%)	9.67 (94.47%)	*
Average Length (bp)	149.6	149.5	149.6	149.6	149.6	*

*, data not available.

Table S8: Statistics of gene structural annotations.

	Feature	Number	Min. length (bp)	Max length (bp)	Median length (bp)	Mean length (bp)
All genes	gene	41,600	55	199,239	2,525	4,492.4
	mRNA	54,195	55	17,507	1,182	1,466.6
	CDS	52,618	153	17,175	864	1,118.7
	exon	300,865	1	7,287	149	264.2
	intron	246,670	4	145,981	394	888.0
Coding genes	gene	40,023	153	199,239	2,730	4,664.1
	mRNA	52,618	153	17,507	1,221	1,506.5
	CDS	52,618	153	17,175	864	1,118.7
	exon	299,256	1	7,287	150	264.9
	intron	246,638	21	145,981	394	888.1
Non-coding genes	rRNA	98	95	3,759	112	581.4
	tRNA	477	60	93	73	74.3
	ncRNA	1,002	55	495	108	121.7

Table S9: BUSCO recovery score of protein coding genes.

BUSCO	Percentage
Complete BUSCOs (C)	93.70%
Complete and single-copy BUSCOs (S)	65.00%
Complete and duplicated BUSCOs (D)	28.70%
Fragmented BUSCOs (F)	3.00%
Missing BUSCOs (M)	3.30%

BUSCO, Benchmarking Universal Single-Copy Orthologs

Table S10: Statistics of coding gene functional annotations.

	Databases	Count	Percentage
Total gene	All	40,023	100%
	Annotated	36,585	91.41%
	Unannotated	3,438	8.59%
Blat	Swiss_Prot	18,860	47.10%
	TrEMBL	29,411	73.50%
	NR	28,608	71.50%
	Pfam	23,864	59.60%
	eggNOG	26,991	67.40%
	GO	25,853	64.60%
	KO	11,102	27.70%
	Unannotated	10,469	26.20%
interProScan	TIGRFAM	2,746	6.86%
	PANTHER	28,802	71.96%
	Gene3D	20,546	51.34%
	SUPERFAMILY	18,853	47.11%
	SignalP_EUK	3,186	7.96%
	ProSitePatterns	4,977	12.44%
	Coils	5,657	14.13%
	Pfam	24,525	61.28%
	PRINTS	3,614	9.03%
	SignalP_GRAM_POSITIVE	2,460	6.15%
	SFLD	200	0.50%
	MobiDBLite	16,275	40.66%
	GO	19,219	48.02%
	ProSiteProfiles	10,734	26.82%
	TMHMM	8,196	20.48%
	ProDom	377	0.94%
	SMART	8,455	21.13%
	CDD	8,783	21.94%
	SignalP_GRAM_NEGATIVE	1,098	2.74%
	Phobius	12,595	31.47%
	PIRSF	1,483	3.71%
	Hamap	677	1.69%
	IPR	26,950	67.34%
	KEGG	1,850	4.62%
	Reactome	3,088	7.72%
	MetaCyc	1,407	3.52%
	Unannotated	3,873	9.68%

Table S11: Statistics of repeat elements.

Order	Superfamily	Number	Length (bp)	Percent (%)	Mean length (bp)
LTR		272,197	277,144,592	42.4119	1,018.18
	Cassandra	245	68,874	0.0105	281.12
	Caulimovirus	1,489	1,514,284	0.2317	1,016.98
	Copia	54,962	32,119,843	4.9154	584.40
	Gypsy	126,460	182,050,172	27.8595	1,439.59
	Pao	69	36,169	0.0055	524.19
	unknown	87,442	61,030,020	9.3395	697.95
LINE		13,962	8,968,898	1.3725	642.38
	CRE	74	23,968	0.0037	323.89
	I-Jockey	204	118,905	0.0182	582.87
	L1	8,745	6,790,123	1.0391	776.46
	L2	1,196	799,016	0.1223	668.07
	R2-Hero	162	107,164	0.0164	661.51
	RTE-BovB	3,581	1,129,722	0.1729	315.48
SINE		8,644	1,341,669	0.2053	155.21
	ID	31	3,773	0.0006	121.71
	tRNA	8,118	1,237,596	0.1894	152.45
	tRNA-RTE	448	85,419	0.0131	190.67
DNA		42,437	17,668,757	2.7039	416.35
	CMC-EnSpm	2,199	944,550	0.1445	429.54
	Kolobok-T2	625	406,502	0.0622	650.40
	Maverick	326	67,746	0.0104	207.81
	MuLE-MuDR	7,598	5,746,242	0.8794	756.28
	PIF-Harbinger	1,847	647,368	0.0991	350.50
	PIF-ISL2EU	714	216,791	0.0332	303.63
	TcMar	703	425,741	0.0652	605.61
	TcMar-Stowaway	1,385	291,387	0.0446	210.39
	Zisupton	716	133,854	0.0205	186.95
	hAT-Ac	8,695	2,796,100	0.4279	321.58
	hAT-Charlie	739	1,696,016	0.2595	2,295.01
	hAT-Tag1	3,570	1,007,393	0.1542	282.18
	hAT-Tip100	3,441	901,894	0.138	262.10
	hAT-hATm	50	14,822	0.0023	296.44
RC		2,499	1,393,956	0.2133	557.81
	Helitron	2,499	1,393,956	0.2133	557.81
Unknown		125,759	32,606,422	4.9898	259.28
rRNA		157	46,587	0.0071	296.73
Satellite		1,465	627,062	0.096	428.03
Simple_repeat		174,773	8,329,452	1.2747	47.66
Low_complexity		26,758	1,301,774	0.1992	48.65
snRNA		190	58,618	0.009	308.52
total		668,841	349,487,787	53.4828	522.53

Table S12: Statistics of LTR-RTs classification in two *Rhododendron* species.

LTR-RTs	Class	*R. molle*	*R. simsii*
all	intact+solo+truncated	32,944	14,334
all	Copia	2,195	2,189
all	Gypsy	4,795	1,785
all	Gypsy|Ogre	1,107	472
all	Gypsy|Retand	2,179	508
all	Unclassified	1,433	1,112
all=intact	Copia|Ale	572	510
all=intact	Copia|Alesia	4	1
all=intact	Copia|Angela	79	153
all=intact	Gypsy|Athila	460	230
all=intact	Copia|Bianca	25	6
all=intact	Gypsy|CRM	135	118
all=intact	Gypsy|Galadriel	12	3
all=intact	Copia|Ikeros	93	77
all=intact	Copia|Ivana	100	73
all=intact	Gypsy|Reina	8	11
all=intact	Copia|SIRE	66	44
all=intact	Copia|TAR	147	186
all=intact	Gypsy|Tekay	404	220
all=intact	Copia|Tork	166	250
intact	intact	3,239	2,096
intact	Copia	1,252	1,300
intact	Gypsy	1,987	796
intact	Gypsy|Ogre	273	122
intact	Gypsy|Retand	695	92
solo	solo	25,954	10,360
truncated	truncated	3,751	1,878
truncated	Copia	943	889
truncated	Gypsy	2,808	989
truncated	Copia|Unclassified	943	889
truncated	Gypsy|Ogre	834	350
truncated	Gypsy|Retand	1,484	416
truncated	Gypsy|Unclassified	490	223

“all” indicates all of the intact, solo, and truncated LTR-RTs; “all=intact” indicates that numbers of all and intact LTR-RTs are equal.

Table S13: Birth and death of long terminal repeat-retrotransposons (LTR-RTs) in two *Rhododendron* species.

	R. molle	***R. simsii***
Intact LTR-RT (*I*)	3,239	2,096
Solo-LTR (*S*)	25,954	10,360
Truncated LTR (*T*)	3,751	1,878
S+*T*	29,705	12,238
S+*T*+*I*	32,944	14,334
All Module Sum	561	389
Filtered scaffold length (bp)	335,000	900,000
Filtered I	3,230	1,880
Filtered S	25,930	9,067
Filtered T	3,748	1,593
Filtered *S/I*	8.03	4.82
Filtered *T/I*	1.16	0.85
Filtered (*S*+*T*)/*I*	9.19	5.67
S/I>3 within Module	267	172
S/I>3 ModuleSum/AllModuleSum	47.59%	44.21%

Table S14: Quantity and proportion of genes in tandem or proximal gene clusters related to pigmentation metabolic pathway.

		R. molle	***R. simsii***
Anthocyanin	ALL	104	125
	PD	15 (14.42%)	9 (7.2%)
	TD	32 (30.76%)	46 (36.8%)
	TD/PD	47 (45.19%)	55 (44%)
Anthocyanin-flavonol shared	ALL	111	139
	PD	17 (15.31%)	11 (7.91%)
	TD	32 (28.82%)	49 (35.25%)
	TD/PD	49 (44.14%)	60 (43.16%)
Flavonol	ALL	90	112
	PD	14 (15.55%)	11 (9.82%)
	TD	30 (33.33%)	42 (37.5%)
	TD/PD	44 (48.88%)	53 (47.32%)
Carotenoid	ALL	52	58
	PD	4 (7.69%)	4 (6.89%)
	TD	8 (15.38%)	6 (10.34%)
	TD/PD	12 (23.07%)	10 (17.24%)
Chlorophyll	ALL	64	54
	PD	7 (10.93%)	2 (3.7%)
	TD	10 (15.62%)	10 (18.51%)
	TD/PD	17 (26.56%)	12 (22.22%)

ALL, all identified genes; TD, tandem duplicated genes; PD, proximal duplicated genes; TD/PD, tandem or proximal duplicated genes.

Table S15: Statistics of flower pigmentation genes annotated in the genomes of *R. simsii* and *R. molle*.

Species	Pigment	Raw enzyme	Raw gene	Filtered enzyme	Filtered gene	Pseudogene enzyme	Pseudogene
R. molle	anthocyanins	15	104	12	93	8	25
	carotenoid	21	52	21	45	2	3
	chlorophyll	23	64	23	62	4	4
	flavonols	8	90	8	83	6	23
R. simsii	anthocyanins	16	125	15	117	8	16
	carotenoid	21	58	21	54	3	3
	chlorophyll	24	54	24	53	2	3
	flavonols	8	112	8	105	4	9

Table S16: Statistics of gene and family number among three TO-GCNs (*R. molle*-specific, *R. simsii*-specific and the consensus TO-GCNs).

		**Consensus TO-GCN**	***R. molle*-specific TO-GCN**	***R. simsii*-specific TO-GCN**
TF+ Pigment enzymatic genes	all	372 (73)	950 (103)	895 (102)
	initial	270 (61)	538 (83)	565 (94)
	terminal	75 (32)	365 (67)	265 (51)
	transitional	27 (17)	47 (29)	65 (32)
TF	all	323 (48)	817 (56)	736 (53)
	initial	239 (41)	476 (49)	471 (49)
	terminal	62 (27)	301 (43)	207 (36)
	transitional	22 (14)	40 (24)	58 (27)
Pigment enzymatic genes	all	49 (25)	133 (47)	159 (49)
	initial	13 (20)	41 (34)	59 (45)
	terminal	6 (5)	21 (24)	26 (15)
	transitional	19 (3)	33 (5)	31 (5)
	anthocyanin	11 (7)	38 (12)	43 (13)
	initial|anthocyanin	31 (4)	62 (12)	94 (12)
	terminal|anthocyanin	5 (4)	17 (8)	28 (9)
	transitional|anthocyanin	6 (1)	8 (2)	19 (2)
	carotenoid	17 (4)	24 (16)	28 (15)
	initial|carotenoid	3 (4)	13 (7)	19 (13)
	terminal|carotenoid	13 (0)	64 (10)	58 (4)
	transitional|carotenoid	7 (0)	22 (1)	29 (2)
	chlorophyll	0 (13)	12 (18)	5 (21)
	initial|chlorophyll	1 (12)	8 (14)	2 (20)
	terminal|chlorophyll	5 (1)	22 (5)	22 (2)
	transitional|chlorophyll	5 (1)	7 (1)	7 (1)
	flavonol	1 (5)	2 (8)	2 (7)
	initial|flavonol	0 (2)	1 (8)	2 (6)
	terminal|flavonol	1 (3)	1 (7)	1 (6)
	transitional|flavonol	3 (2)	3 (3)	2 (2)

Numbers of genes and gene families are separated by a solidus, and the numbers of gene families are in bracket

Table S17: Statistics of mRNA sequencing data for five flowering time points.

Time points	Sample code	Raw reads (Mb)	Raw bases (Gb)	Clean reads (Mb)	Clean bases (Gb)	Clean Q30 (Gb)
Time Point 1	T_1_1	68.789	10.318	66.868 (97.21%)	10.004 (96.95%)	9.668 (94.47%)
	T_1_2	68.408	10.261	66.756 (97.59%)	9.993 (97.38%)	9.675 (94.96%)
	T_1_3	67.909	10.186	66.255 (97.56%)	9.919 (97.37%)	9.608 (94.98%)
	T_1_4	61.773	9.266	60.215 (97.48%)	9.012 (97.26%)	8.793 (95.59%)
	T_1_5	70.4	10.56	68.616 (97.47%)	10.264 (97.19%)	9.929 (94.73%)
Time Point 2	T_2_1	64.748	9.712	62.326 (96.26%)	9.322 (95.98%)	9.156 (95.14%)
	T_2_2	63.272	9.491	61.665 (97.46%)	9.234 (97.29%)	9.029 (95.79%)
	T_2_3	71.833	10.775	69.923 (97.34%)	10.467 (97.14%)	10.205 (95.41%)
	T_2_4	63.15	9.472	61.661 (97.64%)	9.222 (97.36%)	9.021 (95.90%)
	T_2_5	66.918	10.038	65.276 (97.55%)	9.768 (97.31%)	9.514 (95.45%)
Time Point 3	T_3_1	68.958	10.344	67.248 (97.52%)	10.062 (97.28%)	9.728 (94.76%)
	T_3_2	67.457	10.119	65.463 (97.04%)	9.798 (96.84%)	9.439 (94.06%)
	T_3_3	63.482	9.522	61.906 (97.52%)	9.234 (96.97%)	9.046 (95.71%)
	T_3_4	68.823	10.323	67.118 (97.52%)	10.034 (97.20%)	9.756 (95.17%)
	T_3_5	67.179	10.077	65.542 (97.56%)	9.809 (97.34%)	9.554 (95.45%)
Time Point 4	T_4_1	61.451	9.218	59.922 (97.51%)	8.966 (97.27%)	8.776 (95.89%)
	T_4_2	66.382	9.957	64.837 (97.67%)	9.702 (97.44%)	9.390 (94.98%)
	T_4_3	69.022	10.353	67.361 (97.59%)	10.073 (97.29%)	9.771 (95.06%)
	T_4_4	60.813	9.122	59.162 (97.28%)	8.850 (97.01%)	8.663 (95.72%)
	T_4_5	61.555	9.233	59.828 (97.19%)	8.948 (96.91%)	8.710 (95.09%)
Time Point 5	T_5_1	71.274	10.691	69.328 (97.27%)	10.370 (97.00%)	10.055 (94.77%)
	T_5_2	68.463	10.269	66.498 (97.13%)	9.915 (96.54%)	9.707 (95.28%)
	T_5_3	68.443	10.267	66.500 (97.16%)	9.943 (96.85%)	9.657 (94.89%)
	T_5_4	66.603	9.99	64.481 (96.82%)	9.622 (96.31%)	9.330 (94.24%)
	T_5_5	65.918	9.888	63.661 (96.58%)	9.500 (96.08%)	9.285 (94.72%)
Total	*	1663.023	249.452	*	*	*

*, data not available.

Table S18. Statistics of the five versions for genome assembly.

Versions of assembly	Strategy	Assemblied genome size (Mb)	Sequence number	N50	L50	Max. length (Mb)	Gene completeness (%)
V0.1	Corrected by NextDenovo + SMARTDENOVO	697	531	2.9	63	31	*
V0.2	Corrected by NextDenovo + WTDBG2	678	3,734	1.0	167	8.5	*
V0.3	NextDenovo	659	174	11	16	57	*
V1.0	V0.3 + pilon	667	174	11	16	57	93.3%
V1.1	V1.0 + Hi-C + gapclose×2 + nextpolish×3	653	34/25#	45/53#	7/6#	64/68#	93.4%

N50: shortest sequence length at 50% of the genome; L50: smallest number of contigs whose length sum produces N50. *: data not available; # statistics for contigs/scaffolds. Gene completeness was generated by assessment with 1,440 single copy orthologs from the BUSCO embryophyta_odb9 database.

Table S19: Genomic data used for phylogenomic and gene family analyses.

Order	Family	Species	Genes	Scaffold N50	Genome size (bp)	Reference (Digital Object Identifier, DOI)
Ericales	Actinidiaceae	*Actinidia chinensis*	33,044	18,944,233	553,842,477	doi.org/10.1186/s12864-018-4656-3
Ericales	Clethraceae	*Clethra arborea*	31,129	67,174	511,026,369	doi.org/10.1186/s13104-020-05254-4
Ericales	Ebenaceae	*Diospyros oleifera*	30,530	45,442,152	812,355,328	doi.org/10.1093/gigascience/giz164
Ericales	Ericaceae	*Vaccinium macrocarpon*	22,836	37,842,272	490,294,896	doi.org/10.3389/fpls.2021.633310
Ericales	Primulaceae	*Aegiceras corniculatum*	40,727	37,736,876	903,074,775	doi.org/10.1111/1755-0998.13347
Ericales	Ericaceae	*Rhododendron delavayi*	32,938	637,826	695,093,305	doi.org/10.1093/gigascience/gix076
Ericales	Ericaceae	*Rhododendron griersonianum*	38,280	52,925,387	676,814,394	doi.org/10.1111/tpj.15399
Ericales	Ericaceae	*Rhododendron ovatum*	41,392	41,122,356	549,707,122	doi.org/10.1111/pbi.13680
Ericales	Ericaceae	*Rhododendron simsii*	32,999	36,350,743	528,637,147	doi.org/10.1038/s41467-020-18771-4
Ericales	Ericaceae	*Rhododendron williamsianum*	23,548	29,011,353	532,293,122	doi.org/10.1093/gbe/evz245
Ericales	Ericaceae	*Roridula gorgonias*	22,655	46,984	284,227,596	doi.org/10.1186/s13104-020-05254-4
Ericales	Theaceae	*Camellia sinensis*	32,770	218,115,851	3,152,597,298	doi.org/10.1038/s41438-020-0288-2
Cornales	Nyssaceae	*Nyssa sinensis*	36,241	43,339,305	1,001,447,681	doi.org/10.1038/s41597-019-0296-y

Clades, gene numbers, genome properties, and references of 13 reference genomes are shown.

Table S20: Summary of gene family analyses.

Features	Statistics
Number of species	14
Number of genes	459,112
Number of genes in orthogroups	431,542
Number of unassigned genes	27,570
Percentage of genes in orthogroups	94
Percentage of unassigned genes	6
Number of orthogroups	29,747
Number of species-specific orthogroups	5,069
Number of genes in species-specific orthogroups	21,906
Percentage of genes in species-specific orthogroups	4.8
Mean orthogroup size	14.5
Median orthogroup size	11
Number of orthogroups with all species present	7,478
Number of single-copy orthogroups	319
Number of low-copy orthogroups	1,282
Number of genes in *R. molle*	40,023
Number of genes in orthogroups in *R. molle*	37,572
Number of unassigned genes in *R. molle*	2,451
Percentage of genes in orthogroups in *R. molle*	93.9
Percentage of unassigned genes in *R. molle*	6.1
Number of orthogroups containing species in *R. molle*	18,767
Percentage of orthogroups containing species in *R. molle*	63.1
Number of species-specific orthogroups in *R. molle*	395
Number of genes in species-specific orthogroups in *R. molle*	1,361
Percentage of genes in species-specific orthogroups in *R. molle*	3.4

Table S21: Summary of transcription factors annotation.

Family size	TF Family
253	WD40
179	FAR1
164	bHLH
118	MYB
115	NAC
114	ERF
81	C2H2
64	MYB_related
58	Nin-like
57	bZIP
56	WRKY
55	B3
50	GRAS
45	LBD
44	C3H
41	M-type_MADS
38	HD-ZIP
36	G2-like
34	MIKC_MADS
29	TCP; Trihelix
28	Dof
27	GATA
23	ZF-HD
20	HSF; SBP
17	ARF; TALE
15	AP2
13	ARR-B; GeBP
12	HB-other; NF-YB; WOX
11	BES1; NF-YC
10	CO-like; GRF
8	E2F/DP; NF-YA
7	BBR-BPC
6	CAMTA; SRS; YABBY
5	CPP; DBB
4	RAV
3	EIL; HRT-like; Whirly
2	HB-PHD; LSD; NF-X1; S1Fa-like; VOZ
1	LFY; NZZ/SPL; SAP; STAT

Table S22: List of unexpressed enzymatic genes (average TPM = 0) among five time points in the petals.

Species	Enzyme	Pigment	Gene ID
R. molle	acyltransferase	anthocyanin	Rhmol08G0128900
	acyltransferase	anthocyanin	Rhmol04G0089800
	acyltransferase	anthocyanin	Rhmol10G0064800
	F3oGT	anthocyanin	Rhmol04G0115700
	4CL	anthocyanin-flavonol shared	Rhmol10G0289000
	4CL	anthocyanin-flavonol shared	Rhmol10G0289200
	C4H	anthocyanin-flavonol shared	Rhmol03G0152000
	C4H	anthocyanin-flavonol shared	Rhmol05G0315200
	C4H	anthocyanin-flavonol shared	Rhmol07G0142200
	CHS	anthocyanin-flavonol shared	Rhmol09G0044500
	F3H	anthocyanin-flavonol shared	Rhmol01G0339100
	CRTZ	carotenoid	Rhmol08G0001300
	CRTZ	carotenoid	Rhmol09G0130100
	CRTZ	carotenoid	Rhmol12G0085500
	GGPPS	carotenoid	Rhmol10G0159600
	PDS	carotenoid	Rhmol04G0283100
	ZEP	carotenoid	Rhmol06G0024100
	ZEP	carotenoid	Rhmol06G0024700
	CHLG	chlorophyll	Rhmol04G0172500
	GluRS	chlorophyll	Rhmol05G0162200
R. simsii	acyltransferase	anthocyanin	Rhsim03G0189400
	C4H	anthocyanin-flavonol shared	Rhsim01G0166100
	CHI	anthocyanin-flavonol shared	Rhsim03G0120200
	CHS	anthocyanin-flavonol shared	Rhsim09G0036600
	CHS	anthocyanin-flavonol shared	Rhsim09G0048300
	CHS	anthocyanin-flavonol shared	Rhsim09G0053500
	F3′H	anthocyanin-flavonol shared	Rhsim05G0007300
	F3'5'H	anthocyanin-flavonol shared	Rhsim05G0007300
	F3H	anthocyanin-flavonol shared	Rhsim01G0252200
	CRTZ	carotenoid	Rhsim01G0115800
	LCYE	carotenoid	Rhsim01G0069900
	ZEP	carotenoid	Rhsim07G0130400
	GluRS	chlorophyll	Rhsim05G0125800

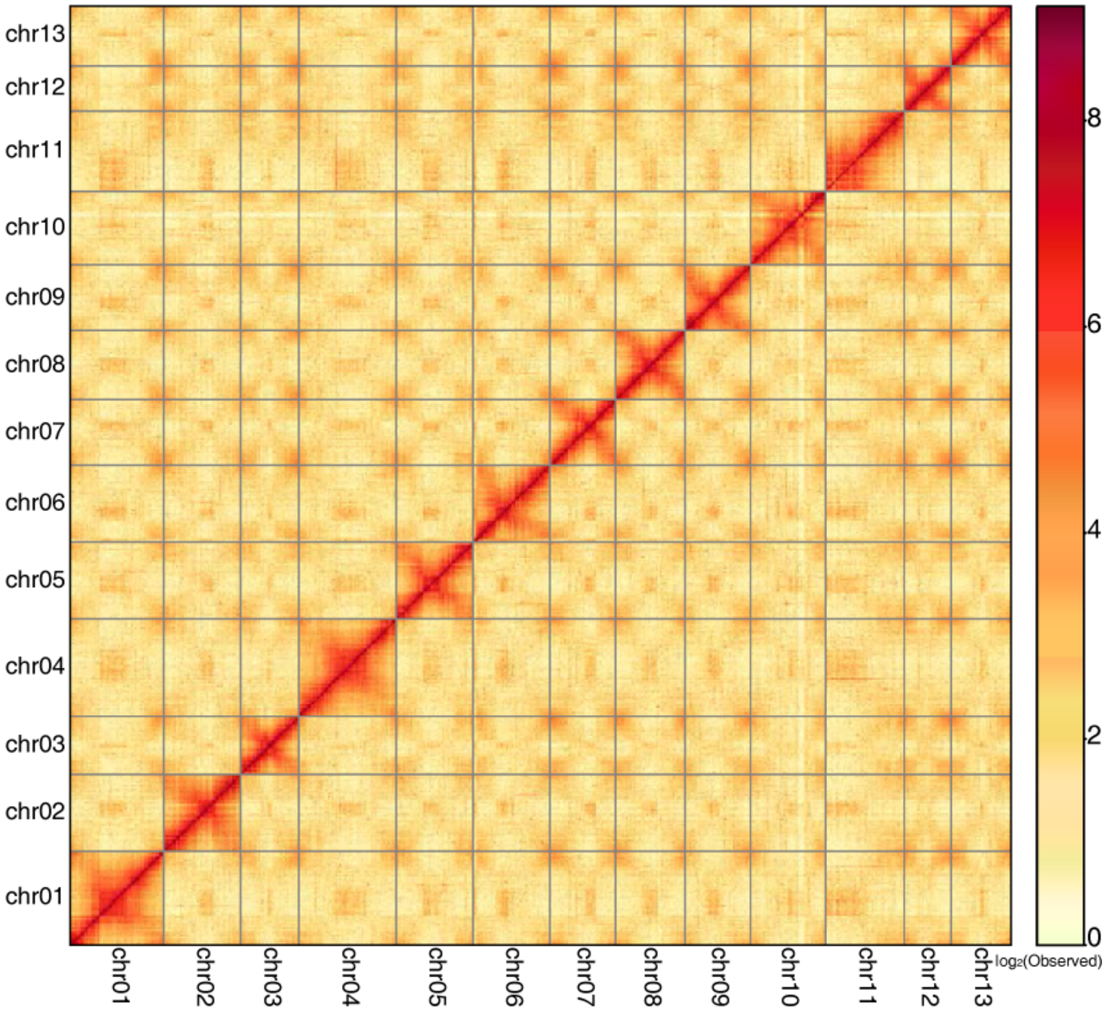

Fig. S1: Genome-wide chromatin interactions with Hi-C data.

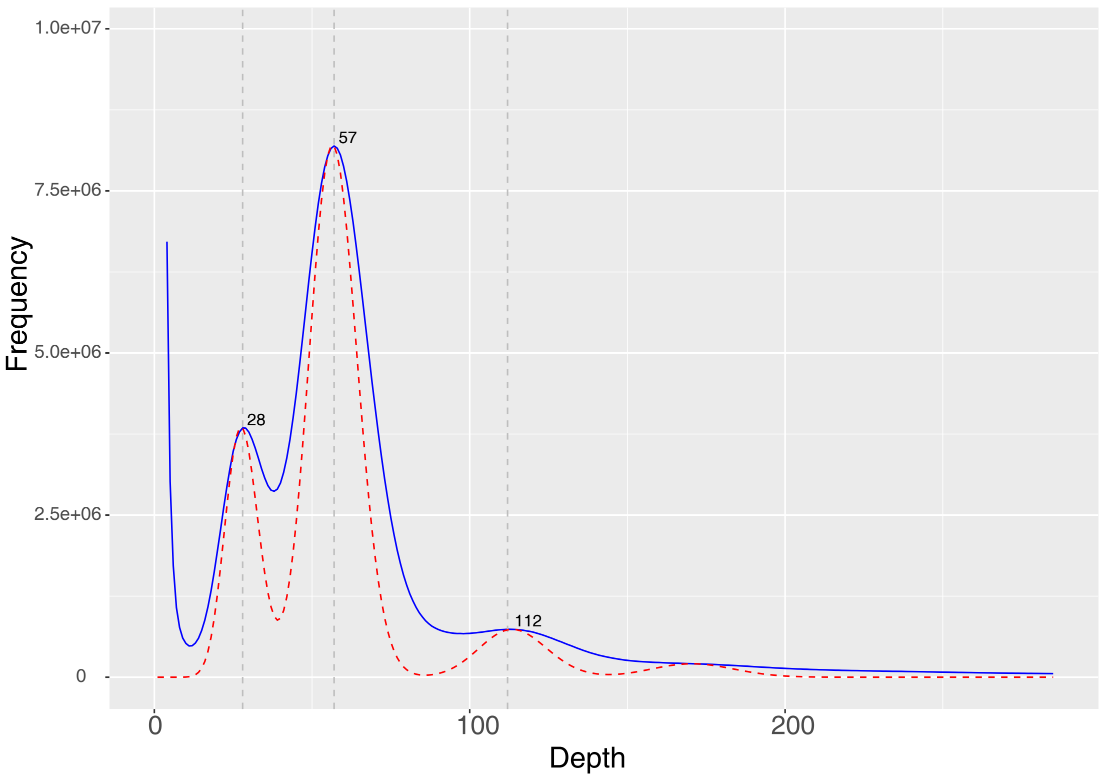

Fig. S2: *k*-mer frequency distribution. *k*=17. Blue solid line for observed *k*-mer frequency distribution, red dash line for fitted model of *k*-mer frequency distribution.

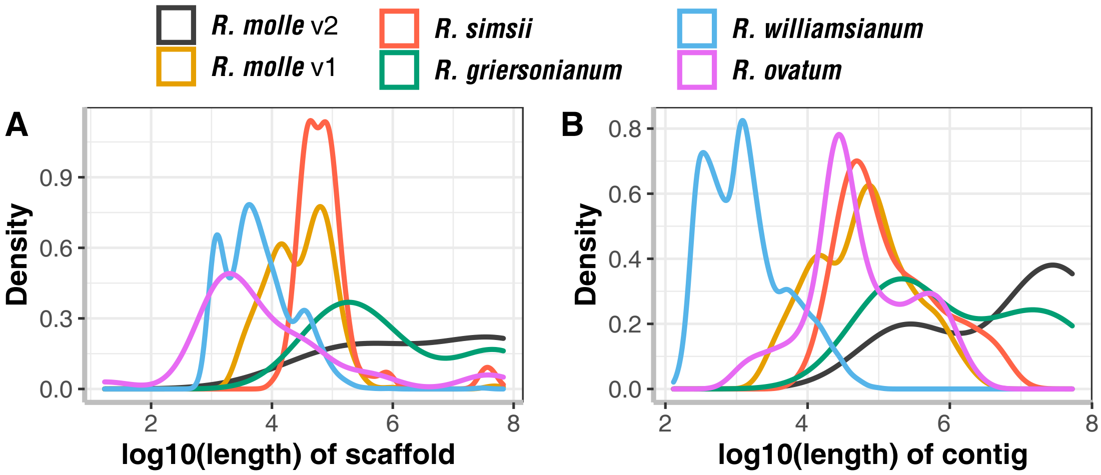

Fig. S3: Length distributions of contig, and scaffold of the assemblies for six *Rhododendron* species using long-read sequencing data. *R. molle* v1 represents the previously reported genome assembly of *R. molle* (Zhou et al., 2022), and *R. molle* v2 represents the genome assembly of *R. molle* in the present study.

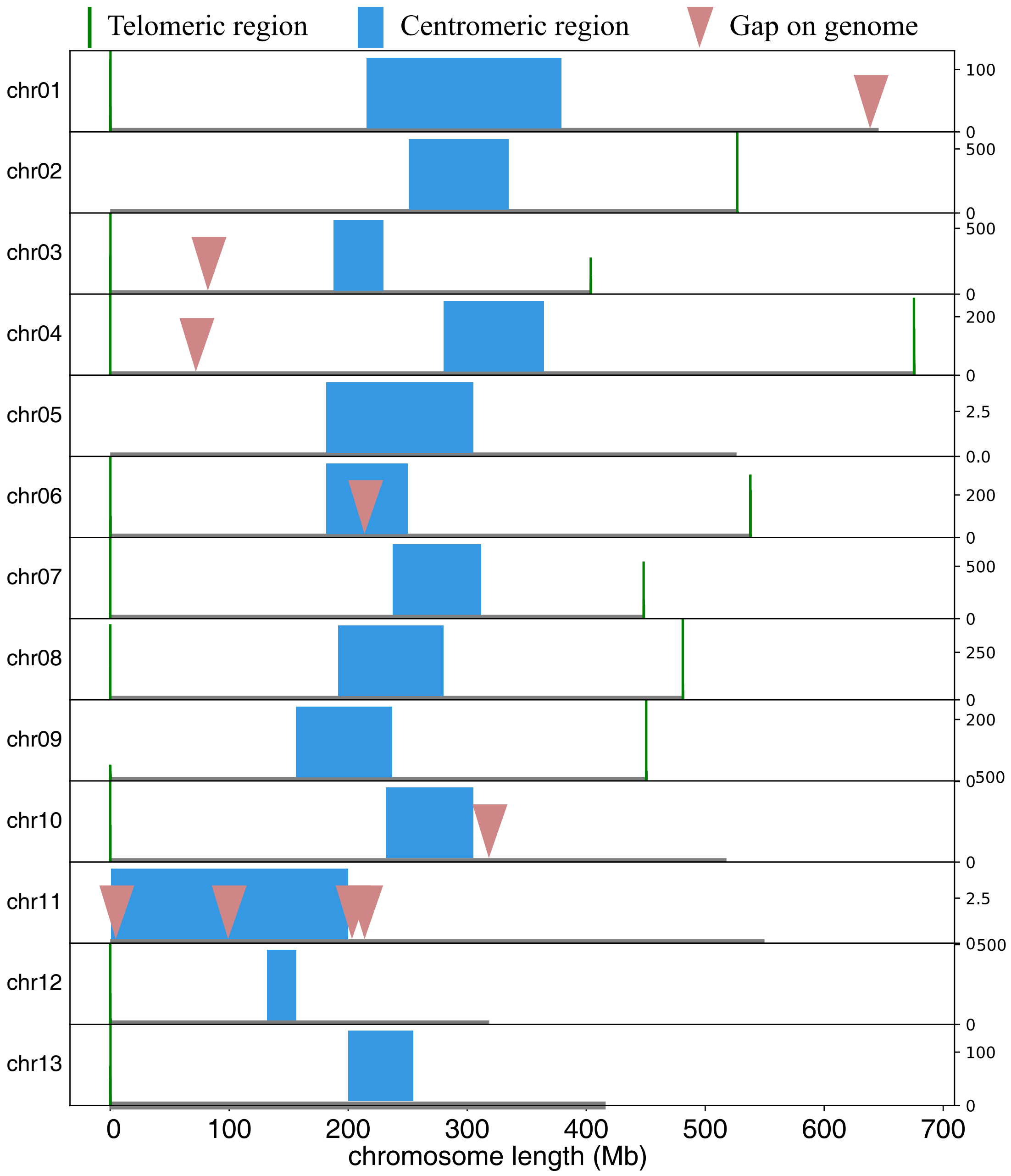

Fig. S4: Positions of sequence gaps, telomeres, and centromeres along the chromosomes.

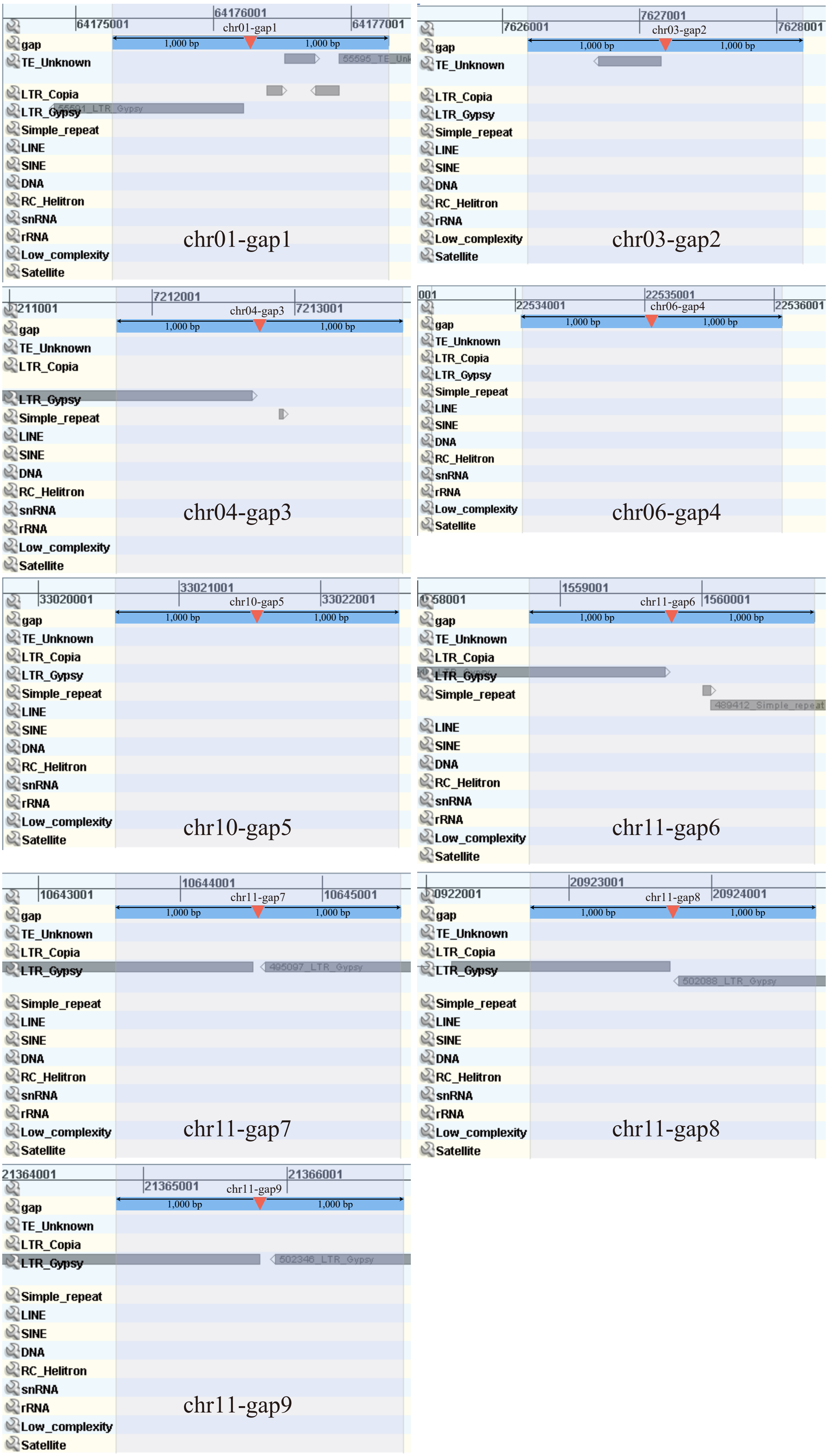

Fig. S5: Zoom-in of each gap and their upstream and downstream regions.

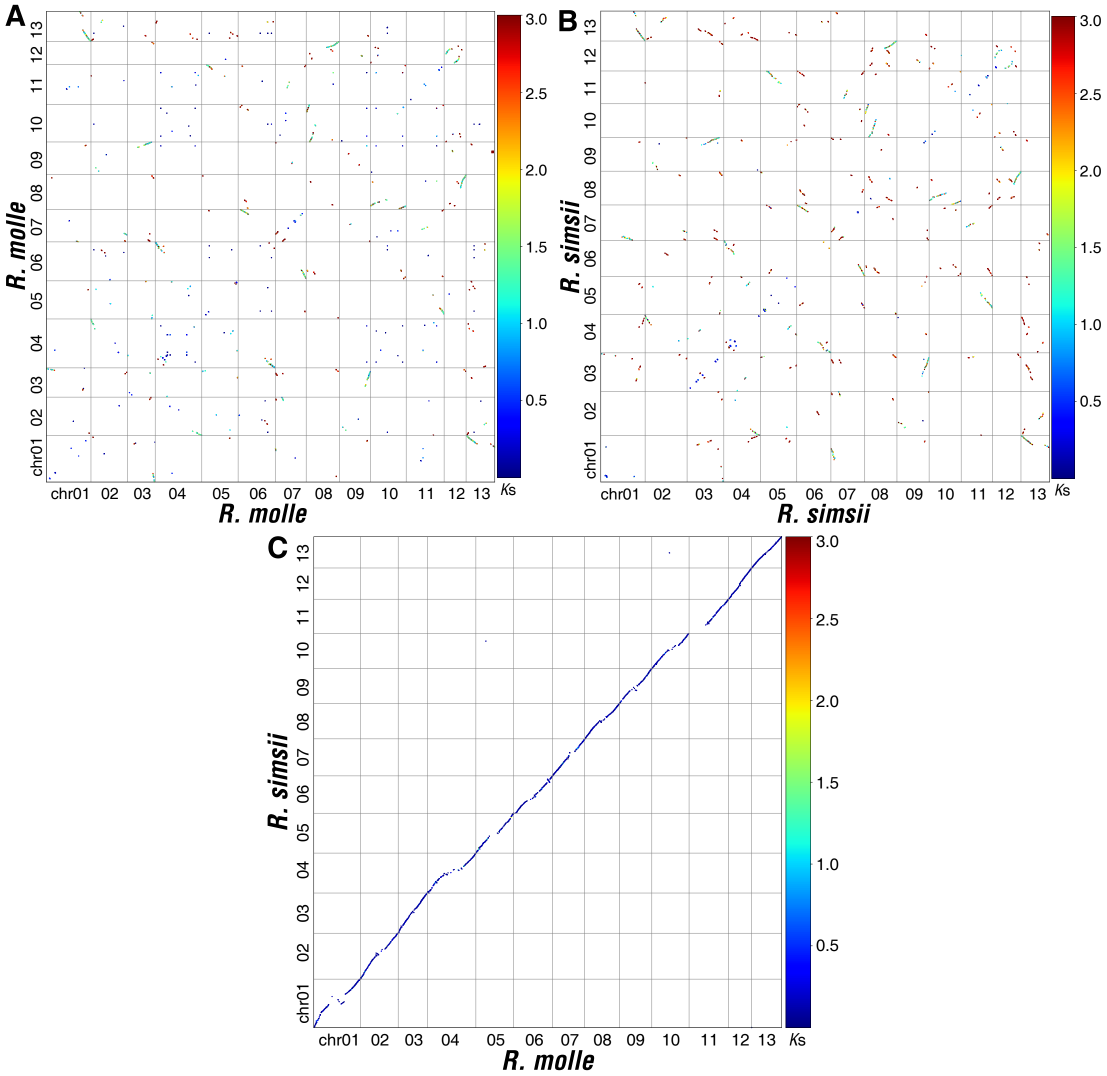

Fig. S6: Interspecific and intraspecific collinearities of the gene orders. The dot plots of paralogous blocks between **A**: *R. molle* and *R. molle*, **B**: *R. simsii* and *R. simsii* and **C**: *R. molle* and *R. simsii*. Chromosome numbers are shown on the horizontal and vertical axes.

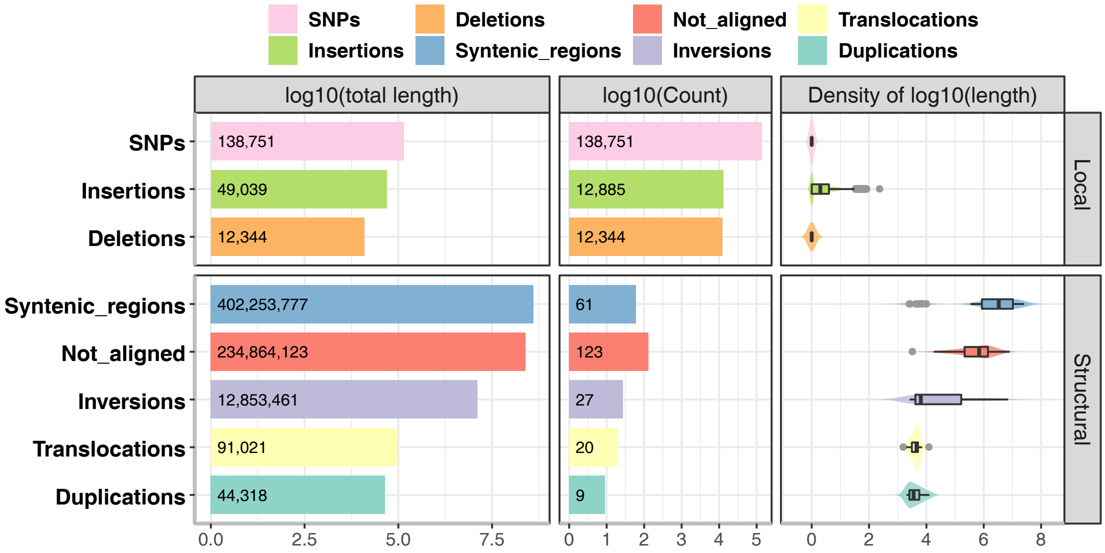

Fig. S7: Length, counts, and length distributions of structural variations and the local sequence differences between *R. molle* and *R. simsii*.

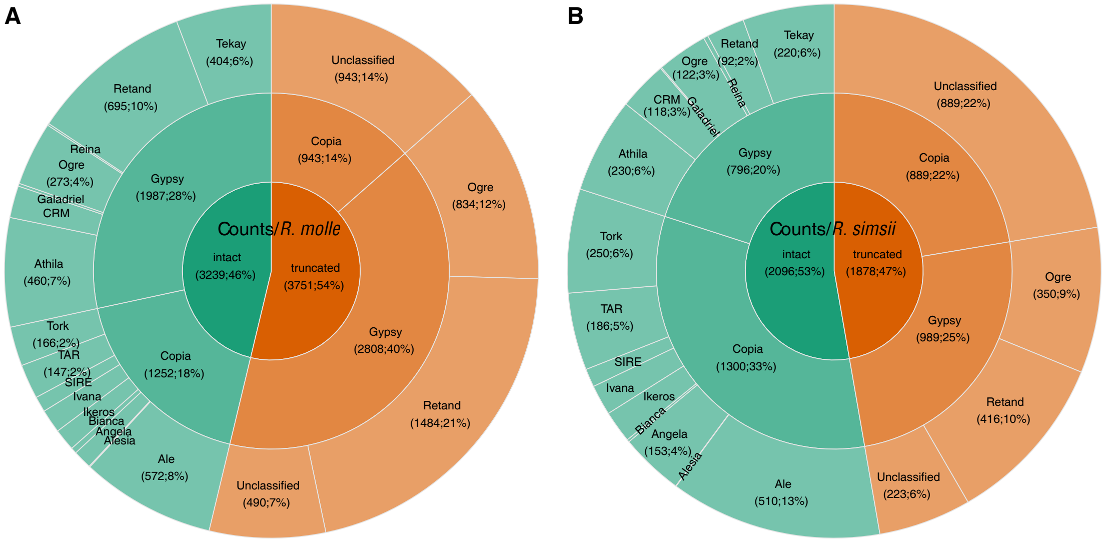

Fig. S8: Count proportions of the LTR-RTs. A, *R. molle*; **B,** *R. simsii*.

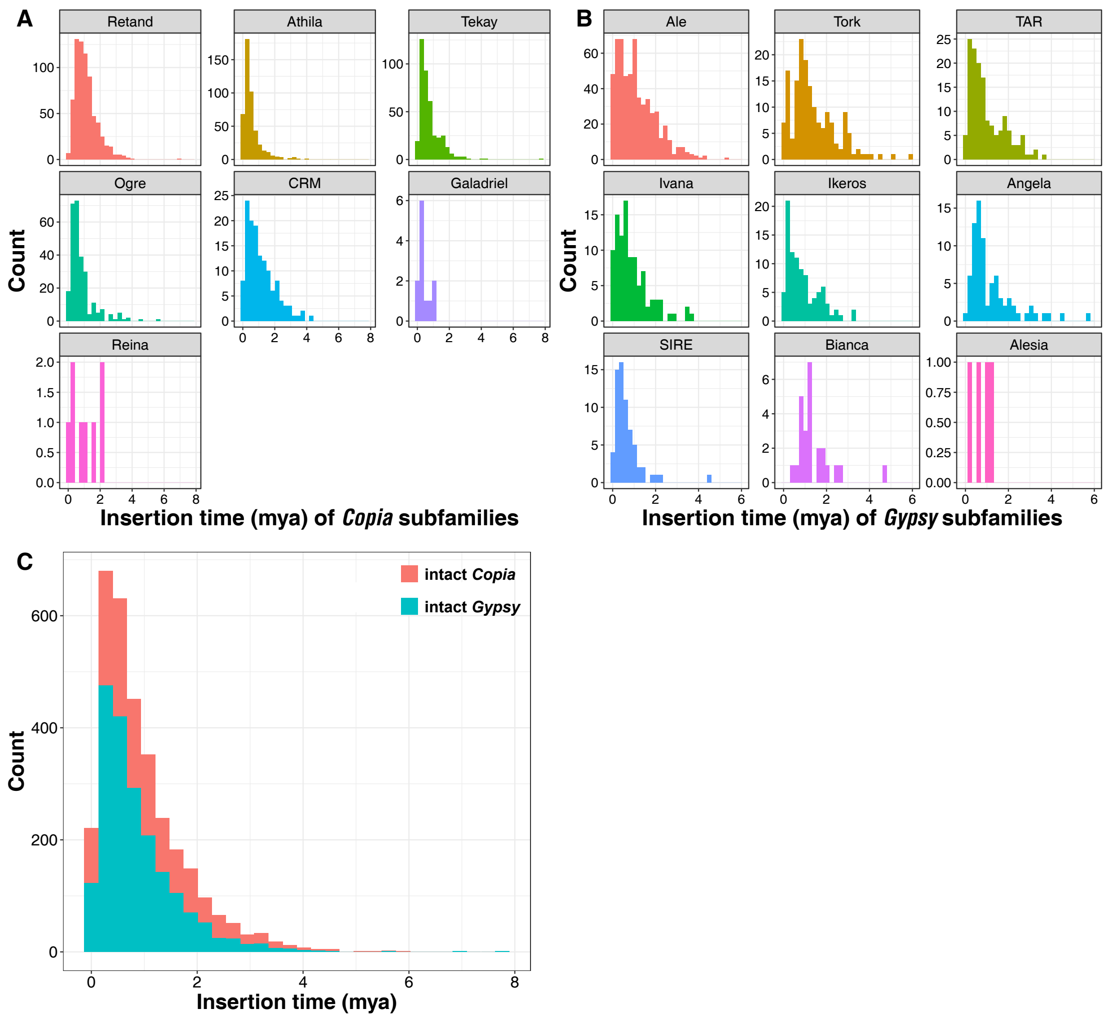

Fig. S9: Insertion time of the *Gypsy* and *Copia* elements. A, families of the *Copia*; **B**, families of the *Gypsy*; **C**, both of *Gypsy* and *Copia*.

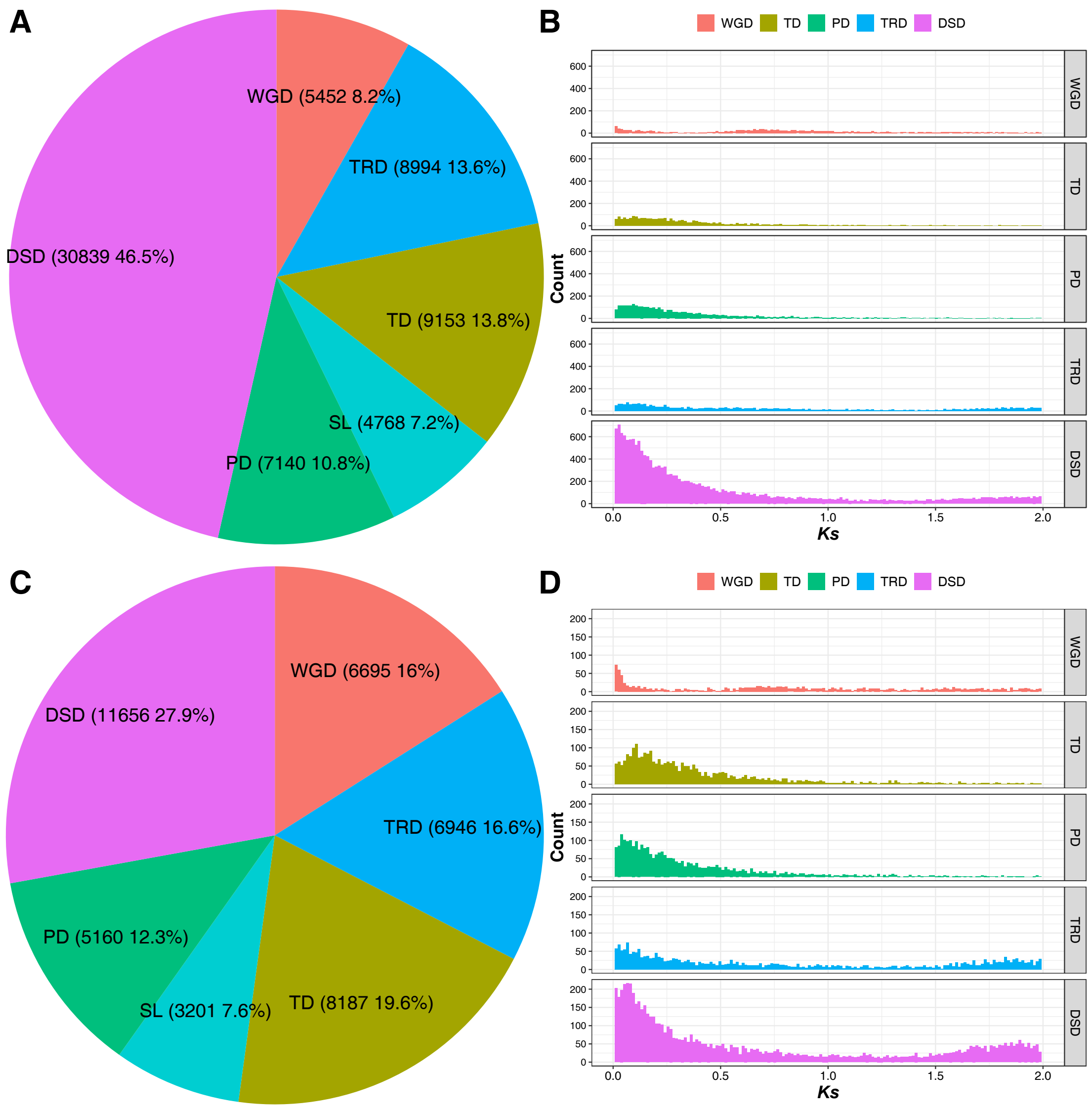

Fig. S10: Gene duplications in *R. molle* and *R. simsii*. **A**, proportions of singletons and duplicates for *R. molle*. **B**, *K*s distribution of the five modes of gene duplications for *R. molle*. **C**, proportions of singletons and duplicates for *R. simsii*. **D**, *K*s distribution of the five modes of gene duplications for *R. simsii*. WGD: whole-genome duplication, TD: tandem duplication, PD: proximal duplication, TRD: transposed duplication, DSD: dispersed duplication.

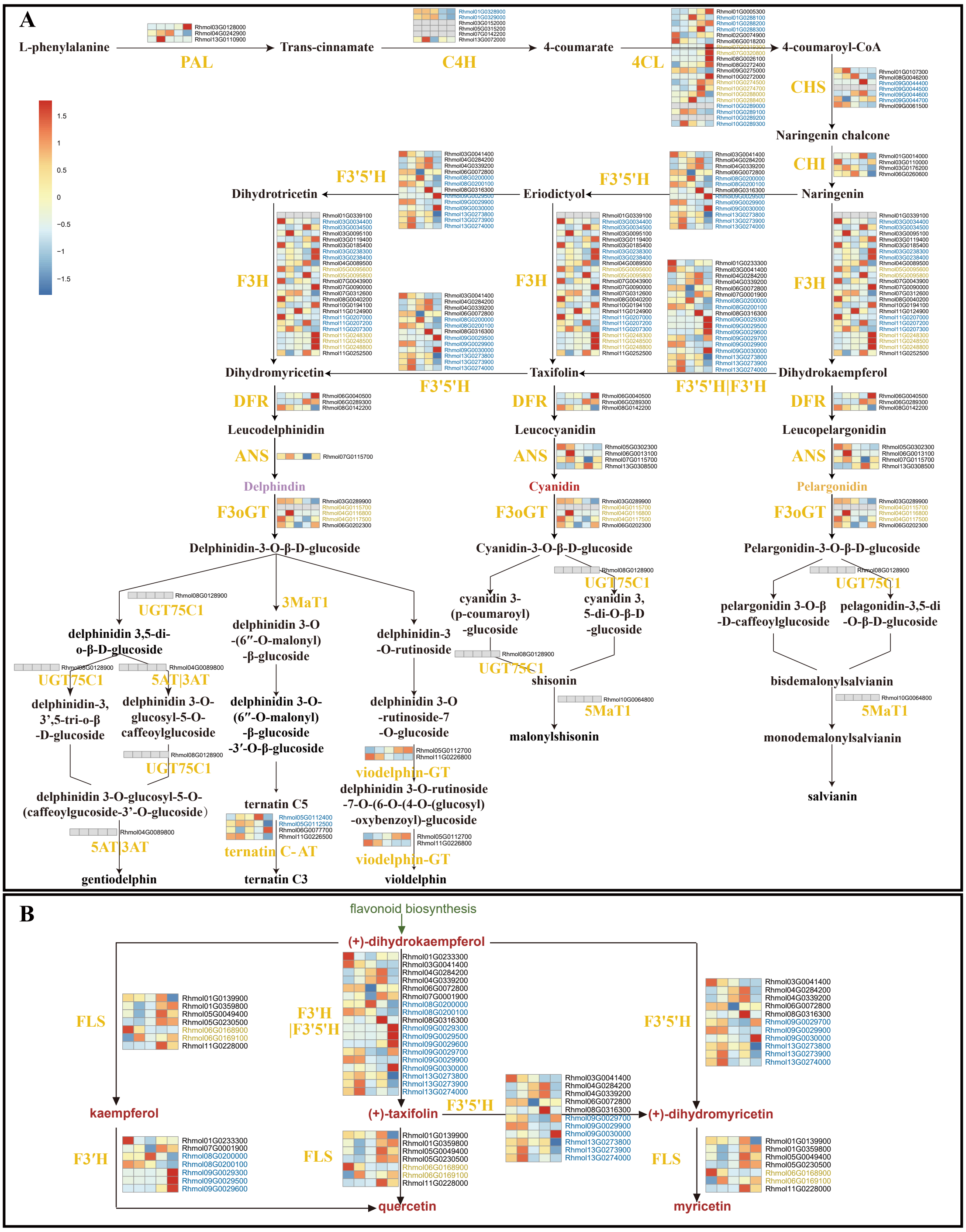

Fig. S11: Metabolic pathway of anthocyanin and flavonol in *R. molle*. A, anthocyanin biosynthesis, **B**, flavonol biosynthesis. Genes identified in tandem clusters are displayed as blue while proximal cluster genes are displayed as brown. Gene expression profile (in normalized TPMs) at different time points of flowering (here T1-T5) are presented in the heatmap alongside gene names. Low to high expression of each gene (z-score) is indicated by a change in color from blue to red. The grey bar represents the zero of the TPM.

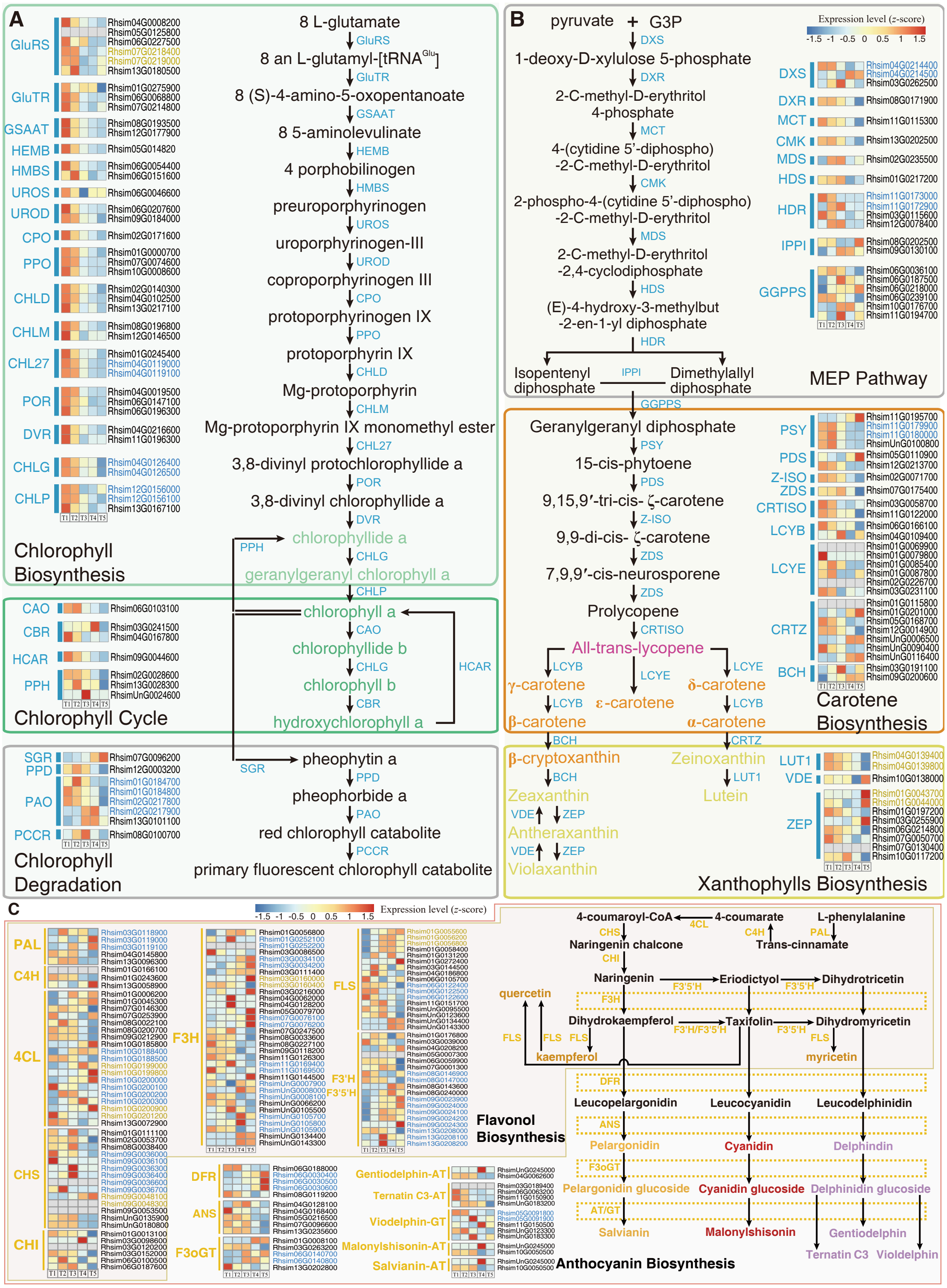

Fig. S12: Metabolic pathways in *R. simsii*. **A**, chlorophyll. **B**, carotenoid. **C**, anthocyanin/flavonol. The pathways on carotenoid and anthocyanin/flavonols were collected from Yang [14], and we rebuilt the chlorophyll metabolic pathway.

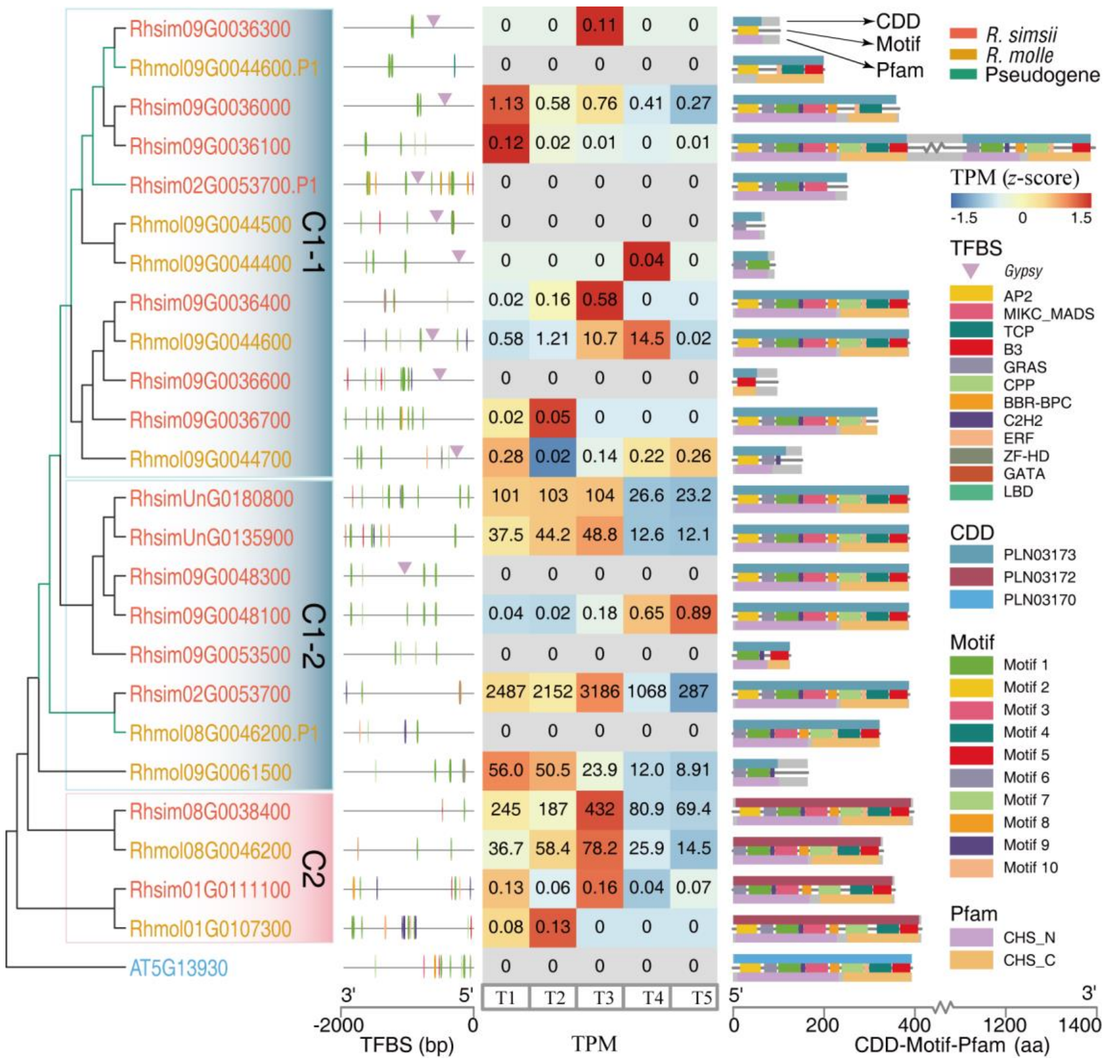

	T1	T2	T3	T4	T5
Rhsim09G0036300	0	0	0.11	0	0
Rhmol09G0044600.P1	0	0	0	0	0
Rhsim09G0036000	1.13	0.58	0.76	0.41	0.27
Rhsim09G0036100	0.12	0.02	0.01	0	0.01
Rhsim02G0053700.P1	0	0	0	0	0
Rhmol09G0044500	0	0	0	0	0
Rhmol09G0044400	0	0	0	0.04	0
Rhsim09G0036400	0.02	0.16	0.58	0	0
Rhmol09G0044600	0.58	1.21	10.7	14.5	0.02
Rhsim09G0036600	0	0	0	0	0
Rhsim09G0036700	0.02	0.05	0	0	0
Rhmol09G0044700	0.28	0.02	0.14	0.22	0.26
RhsimUnG0180800	101	103	104	26.6	23.2
RhsimUnG0135900	37.5	44.2	48.8	12.6	12.1
Rhsim09G0048300	0	0	0	0	0
Rhsim09G0048100	0.04	0.02	0.18	0.65	0.89
Rhsim09G0053500	0	0	0	0	0
Rhsim02G0053700	2487	2152	3186	1068	287
Rhmol08G0046200.P1	0	0	0	0	0
Rhmol09G0061500	56.0	50.5	23.9	12.0	8.91
Rhsim08G0038400	245	187	432	80.9	69.4
Rhmol08G0046200	36.7	58.4	78.2	25.9	14.5
Rhsim01G0111100	0.13	0.06	0.16	0.04	0.07
Rhmol01G0107300	0.08	0.13	0	0	0
AT5G13930	0	0	0	0	0

Fig. S13: Chalcone synthase (CHS) gene family. Phylogenetic relationships, TF binding sites (TFBS) or *Gypsy* insertion in 2kb upstream, Gene expression profile (in normalized TPMs), and conserved domains/motifs of the candidate CHS genes in *R. simsii* and *R. molle* with AT5G13930 as outgroup. Groups I (C1-1/2) and II are indicated by a blue, and red background, respectively. TPM values are labeled in each cell of the heatmap.

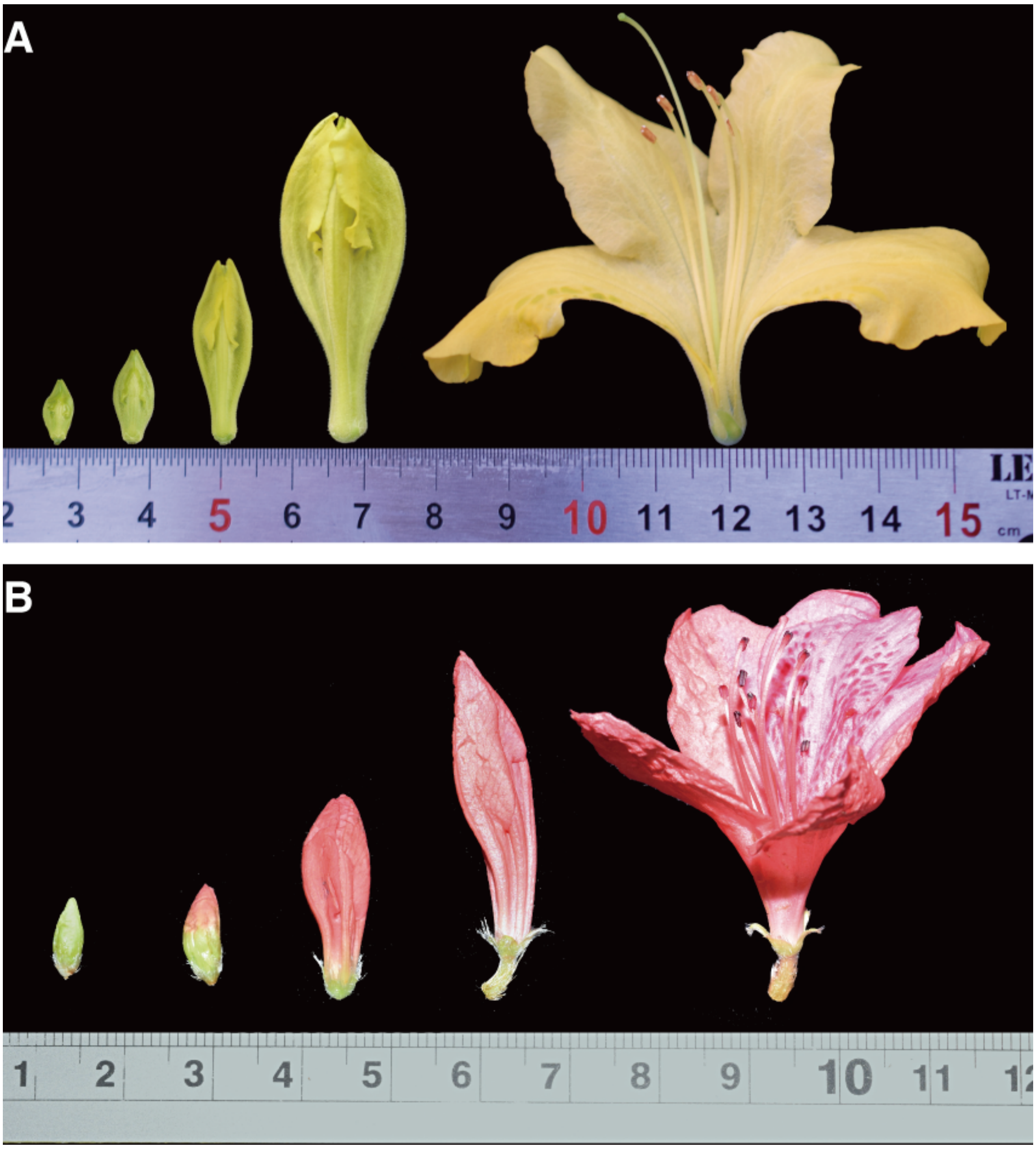

Fig. S14: Five flower developmental time points (T1-T5). A, yellow flowered *R. molle*. **B,** red flowered *R. simsii* (Yang et al, 2020).

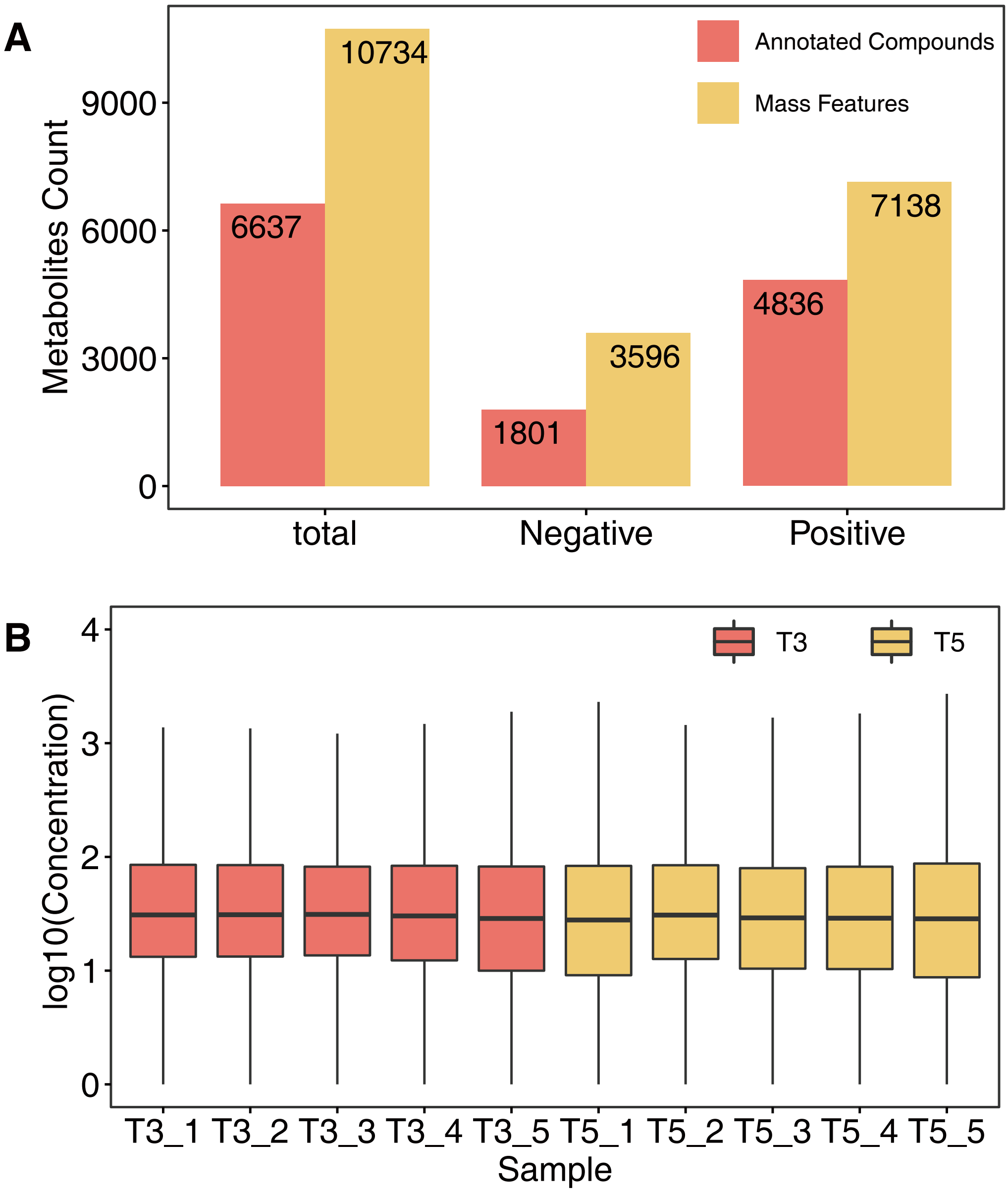

Fig. S15: Metabolites for liquid chromatography–mass spectrometry (LC–MS) at two flower developmental time points (T3 and T5) . A, metabolites count. **B**, distributions of the metabolite concentration.

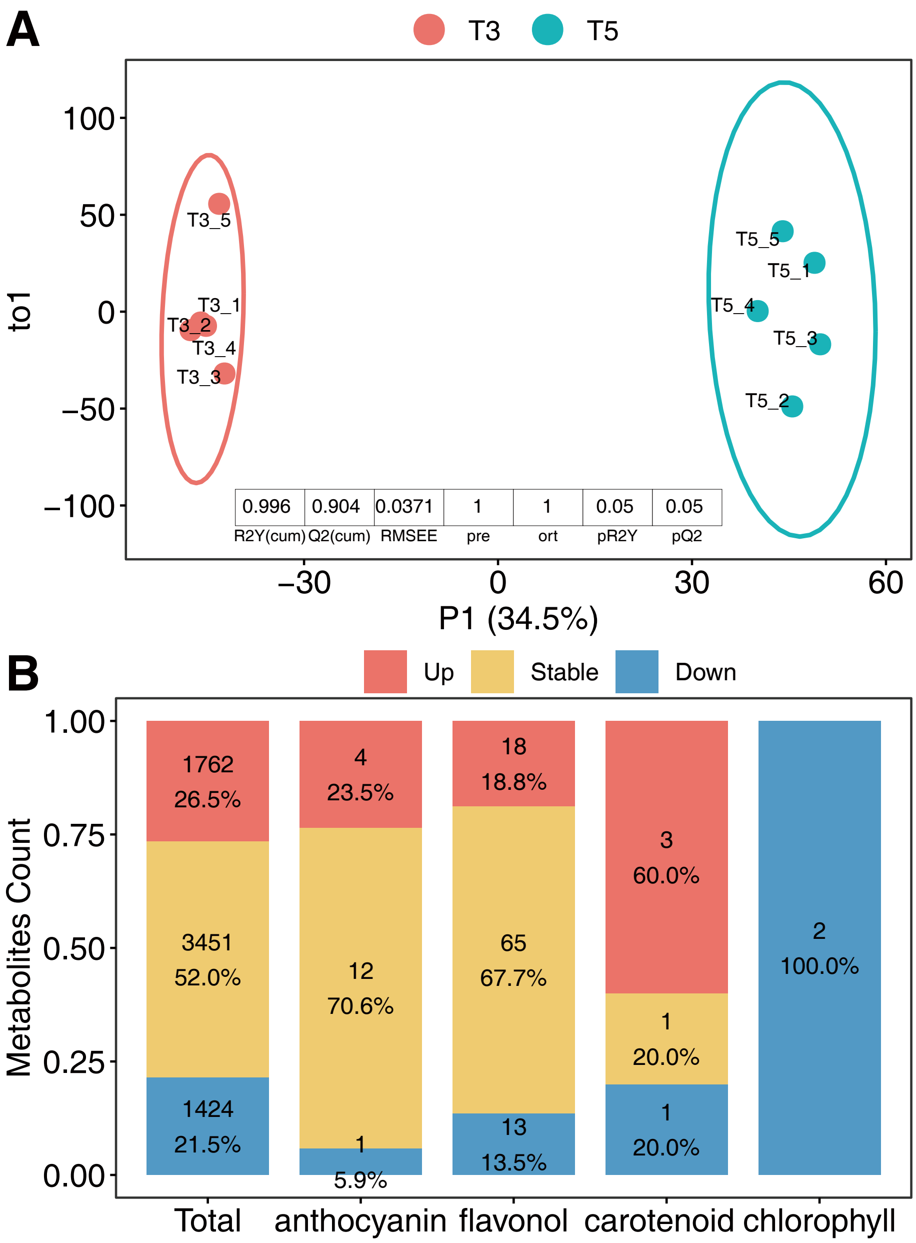

Fig. S16: Differential metabolites between two flower developmental time points (T3 and T5). A, (orthogonal) partial least-squares-discriminant analysis (OPLS-DA) is used to uncover the differential metabolites. Differential metabolites are considered with Variable importance in the projection (VIP) values larger than 1. R2, goodness of fit, is calculated as 1-residual sum of squares (RSS) and the total sum of squares (TSS). Q2, goodness of prediction, is calculated as 1-Predictive residual Error sum of squares (PRESS)/TSS. cum: cumulated. RMSEE: the square root of the mean error between the actual and the predicted responses. pre: correlated (predictive) variation. ort: uncorrelated (orthogonal) variation. **B**, barplot shows the counts of the differential metabolites.

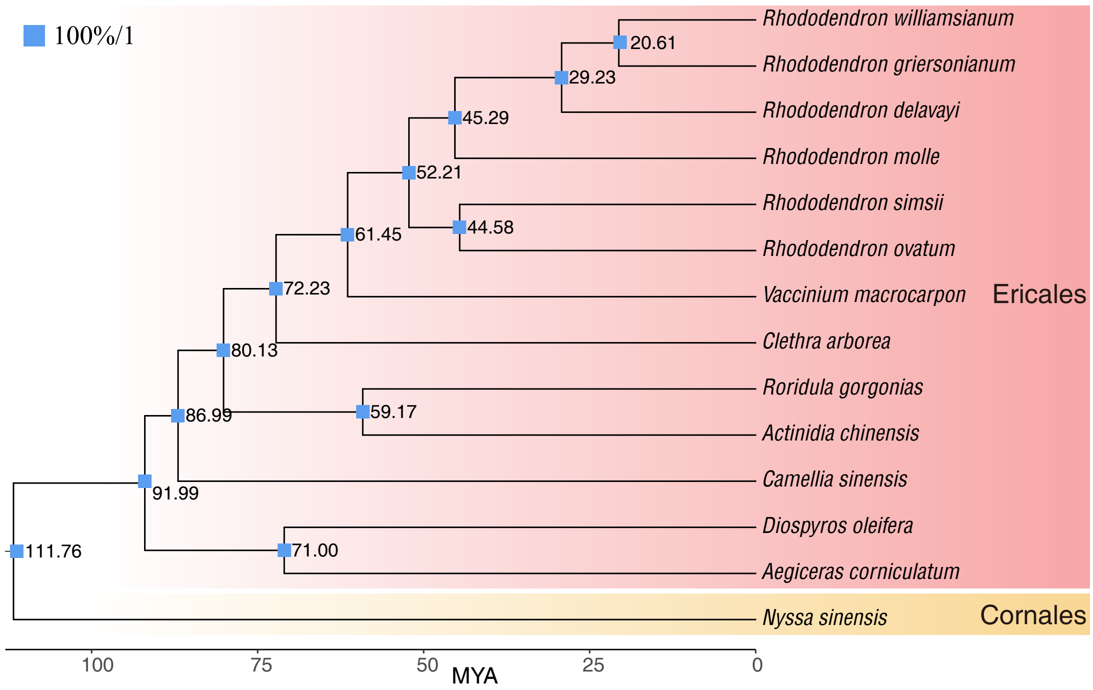

Fig. S17: Genome evolutionary history. Chronogram showing divergence times in ericales and cornales. Blue squares represent support of 100% for each node in both concatenated and coalescent-based phylogenetic trees. The left percentage (100%) is the bootstrap support value in concatenated-based tree, and the right number (1) is the local posterior probability in coalescent-based tree.

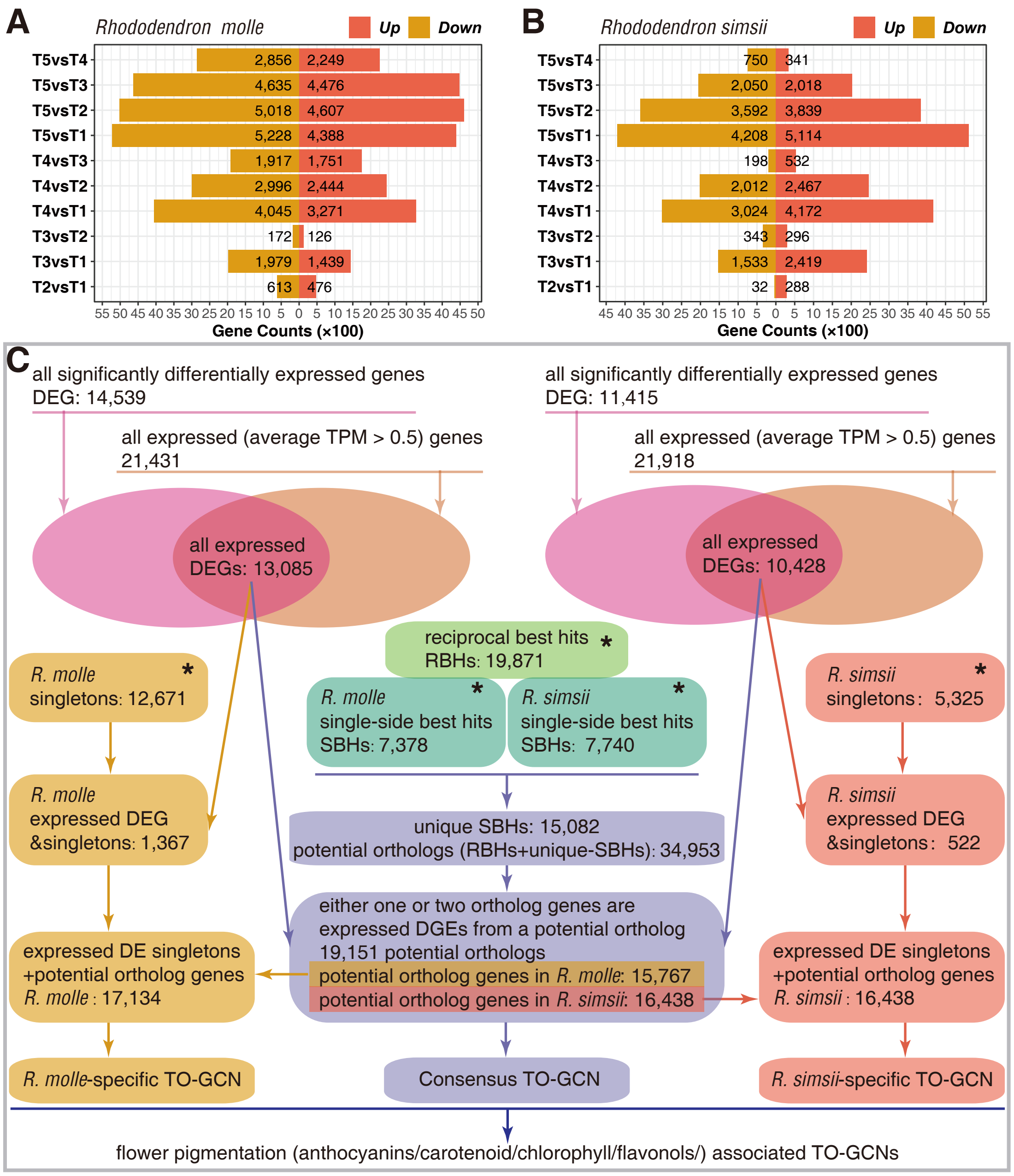

Fig. S18: Details on gene co-expression networks. **A**, the significantly differentially expressed genes (DEGs) between any two points among the five flowering time points in *R. molle*. **B**, DEGs between any two points among the five flowering time points in *R. simsii*. **C**, gene filtering pipeline for TO-GCNs construction. The asterisks represent outputs from GeneTribe .
