# Supplementary material for: Gapless genome assembly of azalea and multi-omics investigation into divergence between two species with distinct flower color
Source: Hortic Res. 2022 Oct 26;10(1):uhac241. doi: 10.1093/hr/uhac241 (PMC9832866; doi:10.1093/hr/uhac241)
Supplement: Web_Material_uhac241 [file web_material_uhac241.zip › ResponseFigures.docx]

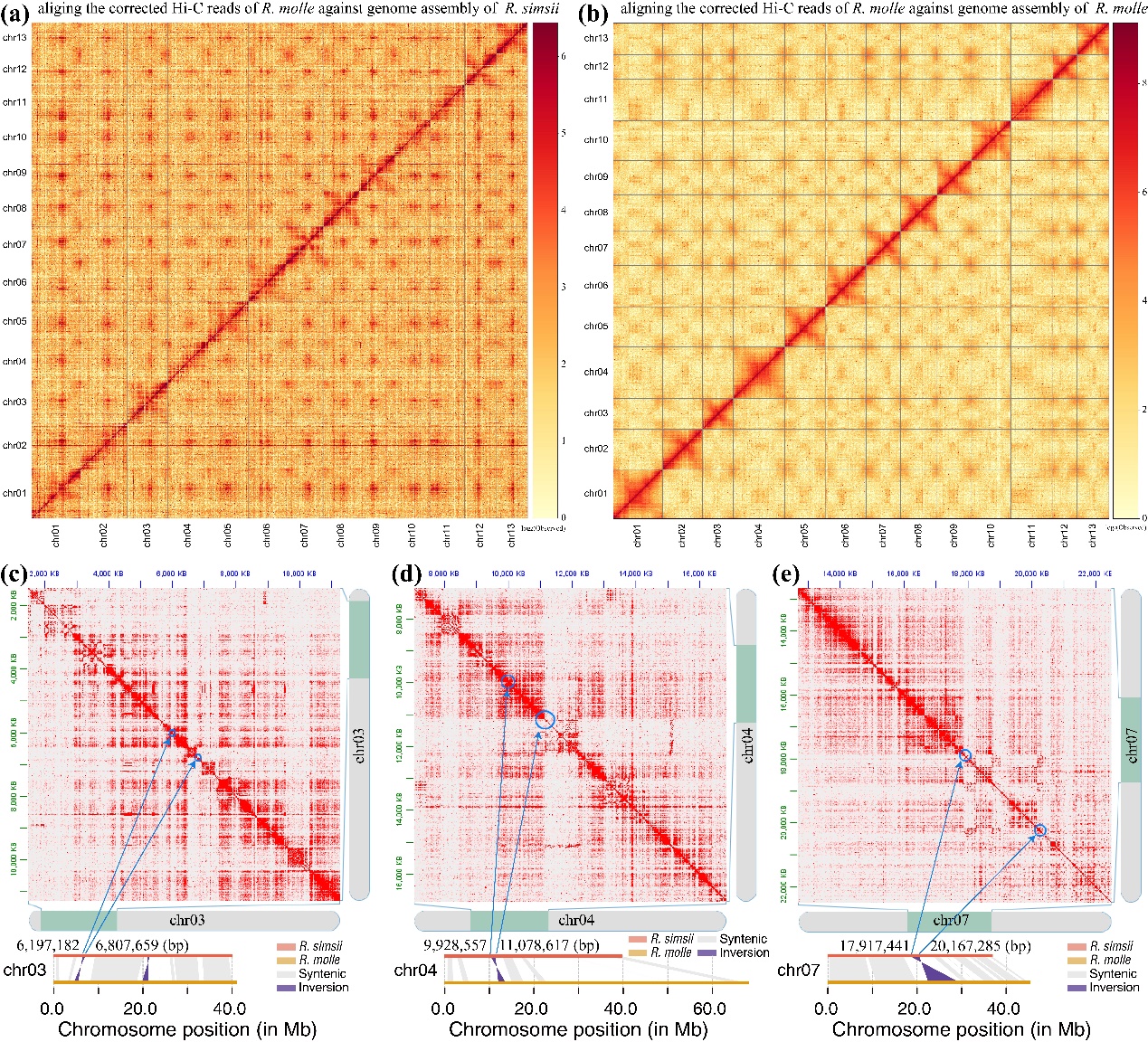


**Response Figure 1.** Validation of the genome rearrangements with *R. simsii*. (a) the Hi-C contact map by aligning the corrected Hi-C reads from *R. molle* against the genome assembly of *R. simsii*. (b) the Hi-C contact map by aligning the corrected Hi-C reads from *R. molle* against the genome assembly of *R. molle*. (c)-(e) validation of large inversions in chromosome chr03 (c), chr04 (d), and chr07 (e) based on Hi-C data. In all c-d, the upper heatmap shows a chromatin interaction maps by aligning Hi-C data from *R. molle* against the *R. simsii*. The lower panel shows a diagram of the inversion region indicated by the purple hourglass.


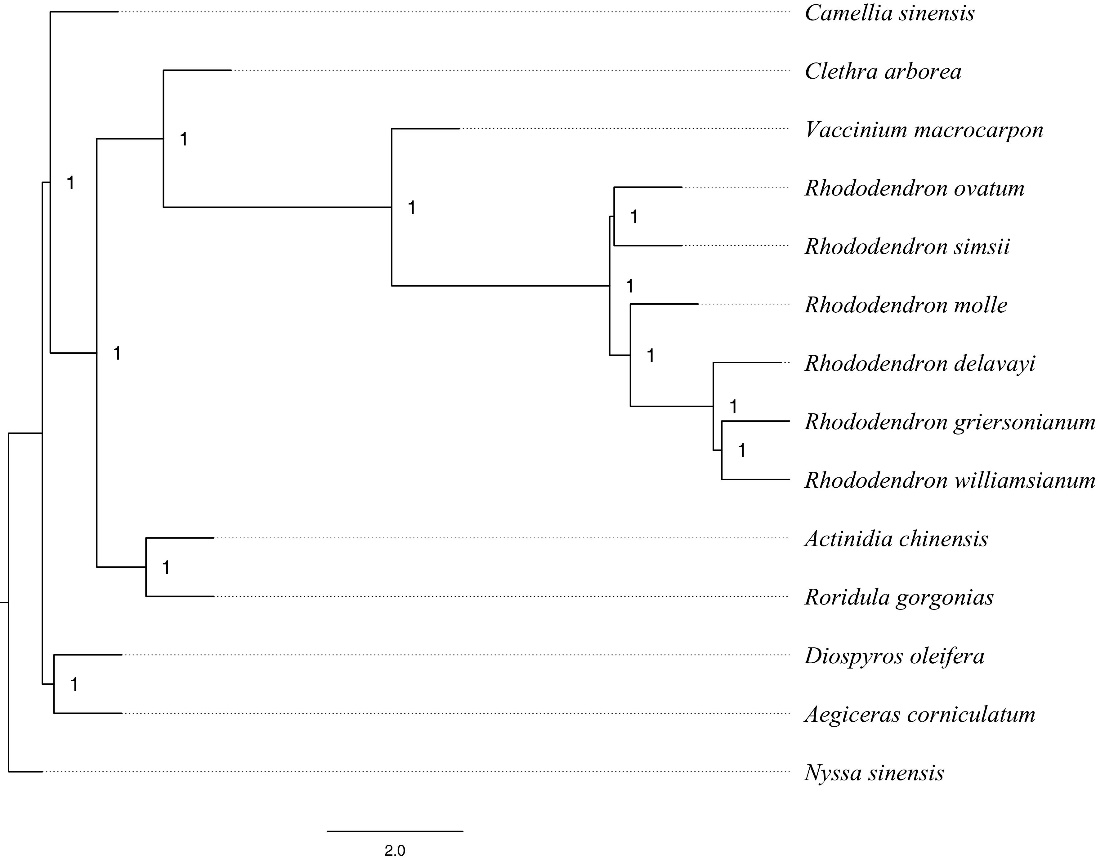


**Response Figure 2.** The coalescent-based phylogenetic inference.
